# Annotated checklist of the land snail fauna from southern Cambodia (Mollusca, Gastropoda)

**DOI:** 10.3897/zookeys.948.51671

**Published:** 2020-07-13

**Authors:** Chirasak Sutcharit, Phanara Thach, Samol Chhuoy, Peng Bun Ngor, Ekgachai Jeratthitikul, Warut Siriwut, Ruttapon Srisonchai, Ting Hui Ng, Arthit Pholyotha, Parin Jirapatrasilp, Somsak Panha

**Affiliations:** 1 Animal Systematics Research Unit, Department of Biology, Faculty of Science, Chulalongkorn University, Bangkok 10330, Thailand Chulalongkorn University Bangkok Thailand; 2 Inland Fisheries Research and Development Institute (IFReDI), Fisheries Administration, No. 86, Norodom Blvd., PO Box 582, Phnom Penh, Cambodia Inland Fisheries Research and Development Institute Phnom Penh Cambodia; 3 Wonders of the Mekong Project, c/o IFReDI, No. 86, Norodom Blvd., PO Box 582, Phnom Penh, Cambodia Wonders of the Mekong Project Phnom Penh Cambodia; 4 Animal Systematics and Molecular Ecology Laboratory, Department of Biology, Faculty of Science, Mahidol University, Bangkok 10400, Thailand Mahidol University Bangkok Thailand; 5 Department of Biology, Faculty of Science, Khon Kaen University, Khon Kaen 40002, Thailand Khon Kaen University Khon Kaen Thailand; 6 Lee Kong Chian Natural History Museum, Faculty of Science, National University of Singapore, 2 Conservatory Drive, Singapore 117377, Republic of Singapore National University of Singapore Singapore Singapore

**Keywords:** Biodiversity, conservation, Indochina, limestones, systematics

## Abstract

Prior to this study, few collections and records were made of the land snails in Cambodia and the historical taxa had never been reviewed. Herein a report on the land snail diversity based on specimens collected recently from karstic and non-karstic areas in southern Cambodia is provided. This checklist presents 36 species of land snails (two Neritimorpha, six Caenogastropoda, and 28 Heterobranchia). Illustrations and brief taxonomic notes/remarks are provided for every species. We also described *Georrisa carinata* Sutcharit & Jirapatrasilp, **sp. nov.** based on some distinct shell morphological characters. Since the first descriptions during the colonial period in the nineteenth century, some land snail species (e.g., *Trichochloritis norodomiana*, *Durgella russeola*, *Anceyoconcha siamensis obesula***comb. nov.**, *Anceyoconcha chaudoensis***comb. nov**., and *Succinea tenuis*) have not been reported subsequently. This probably reflects a lack of knowledge concerning land snail biodiversity in this country. To our knowledge, this is the first comprehensive survey of land snails in southern Cambodia. A need for more field research and systematic revision of the land snails in this interesting region is also highlighted and demonstrated.

## Introduction

Cambodia forms a part of the Indo-Chinese sub-region of the Indo-Burma biodiversity hotspot (Myers et al. 2000, Wikramanayake et al. 2001, Bain and Hurley 2011, Tordoff et al. 2012). Its terrain mostly consists of low-lying plains and the Mekong Delta and is flanked by the Cardamom Mountains in the west and the Annamite chain in the east (Gupta 2005). As with other parts of Indochina, Cambodia has lost most of its forest areas through changes in land use in the past six decades (Davis et al. 2015, Tsujino et al. 2019), resulting in decreased or irreplaceable losses of biodiversity (Hughes 2017). There is an urgent need to implement conservation action to protect the known fauna and flora of the country, as well as conduct intensive research to reveal species that are not known to science (Sodhi et al. 2010).

The inventory of the Cambodian fauna, primarily for the vertebrates and insects, has been increasing and rapidly improved with field surveys in recent years, e.g., for freshwater fish (Hartmann et al. 2013, So et al. 2019), herpetofauna (Neang et al. 2015, Geissler et al. 2019), dragonflies (Kosterin 2012, Kosterin et al. 2012, Kosterin and Chartier 2014), aquatic Hemiptera (Zettel et al. 2017), beetles (Freitag et al. 2018, Jocque et al. 2019), bees (Ascher et al. 2016), and ants (Hosoishi et al. 2012, 2015). Knowledge of a handful of other invertebrate fauna has also been accumulated, e.g., rotifers (Sor et al. 2015), crustaceans (Naruse et al. 2014), and millipedes (Likhitrakarn et al. 2015). Without doubt, many other major groups of invertebrates remain to be explored including the terrestrial snails (Hun et al. 2019). Malacological research in Cambodia can be traced back to the 19^th^ century, but studies have been temporally and spatially sporadic. However, a small number of local investigations have been undertaken in the past decade (Vermeulen et al. 2007, 2019a, b).

The earliest land snail collections in Cambodia were made by the French naturalist Henri Mouhot from the mountainous areas of eastern and southwestern Cambodia during the mid-1800s (Mouhot 1864a, b). In the colonial period of the late 19^th^ to early 20^th^ century, the most prominent land snail collections were carried out by the well-known French explorer Augustus Pavie. He traveled to most parts of Cambodia and accumulated huge collections of natural history objects (Pavie 1904, see also Inkhavilay et al. 2019). The Pavie collections of land snails were then studied and published by Crosse and Fischer (1876), Morlet (1883, 1885, 1886, 1889, 1890), Rochebrune (1881a, b) and some others. Later, a list of 84 taxa of the land snail fauna of Cambodia was compiled and listed in the “Mission-Pavie”, the significant book series by Fischer and Dautzenberg (1904). Some sixty years later, small collections of land snails from southern Cambodia and Vietnam were collected by the geologist, Edmund Saurin and studied by van Benthem Jutting (1962). The species list of molluscan fauna from Cambodia was reviewed by Fischer (1973a–c). The endemic and monotypic slug, *Cambodiparmarion doroshenkoi*, was described from the south of Cambodia (Kuznetsov and Kuzminykh 1999) and Vermeulen et al. (2007, 2019a, b) reported land snails from south Cambodia and Vietnam, mainly focusing on Mekong Delta limestone in the southwest and the foothills of the southern Annamite range in the northeast. In addition, Hun et al. (2019) discovered the giant land snail *Bertia cambojiensis* in Cambodia for the first time. It is clear that the land snails in southern Cambodia remain poorly known (Hun et al. 2019, Vermeulen et al. 2019a, b).

**Figure 1.**
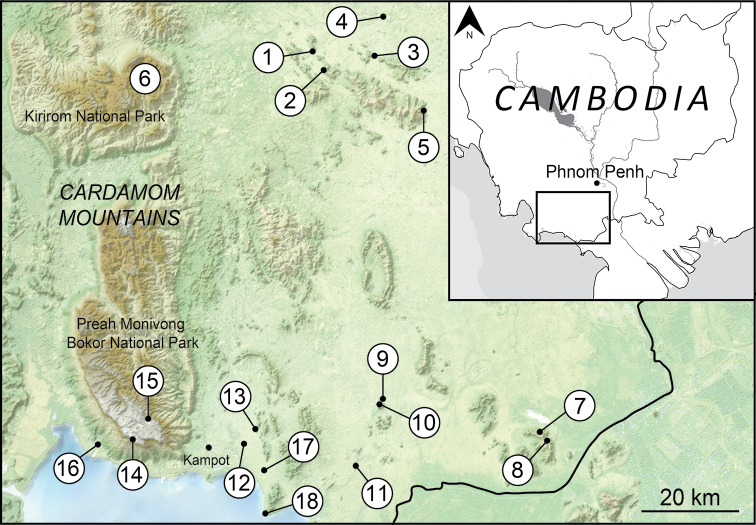
Map of the Kingdom of Cambodia showing the collection localities. The numbers indicate the collection sites which correspond to Table 1 and in the material examined sections.

Southern Cambodia (Fig. 1) represents an interesting biogeographic transition zone between the Cardamom Mountain Ranges in the west, Mekong Delta limestones in the southwest, and the foothills of the southern Annamite range in the northeast (Bain and Hurley 2011, Geissler et al. 2015). Two ecoregions are recognised within this area: the Cardamom Mountains Rain Forests [IM0106] and the Southern Annamites Montane Rain Forests [IM0152] ecoregions (WWF 2019a, b). The aim of our study was therefore to contribute to the filling of a knowledge gap on more land snails from karstic and associated habitat types. Herein, we present the record of land snails collected in September 2019 in the limestone hills, sandstone forest and reserved forest of Kirirom National Park and Preah Monivong Bokor National Park in southern Cambodia.

## Materials and methods

### Field surveys

The survey sites were chosen to cover the main habitat types and the localities are shown in Figure 1 and Table 1. Field surveys were conducted during the day using the encounter survey technique (Crump and Scott 1994). Empty shells were collected by hand. Living snails were searched for in various habitats/micro-habitats such as tree vegetation, decaying logs and leaf litter on forest floor by CS, PT, EJ, WS, RS, TNG, and AP. Living specimens encountered were photographed prior to euthanasia using the 2-step method (AVMA 2013), and these were subsequently fixed and preserved in 70% ethanol.

**Table 1.** Locations and geographical coordinates of sampling sites of terrestrial snails within the southern provinces of Cambodia.

No.	Province	Locality code and name	Latitude / Longitude
1	Kampong Speu	C028-Mountain near Phnom Prak Sombo Pagoda, Tang Sya, Phnum Sruoch District	11°23'53.9"N, 104°23'03.8"E
2		C029-Mountain near Phum Krang Ponley, Khum Kiri Voan, Phnum Sruoch District	11°21'43.90"N, 104°24'14.44"E
3		C031-Phnom Cheal Pagoda, Khum Skuh, Samraong Tong District	11°23'11.81"N, 104°30'34.96"E
4		C032-Bridge, Stoeng Prek Thnaot River, Krong Chbar Mon	11°27'40.15"N, 104°31'43.47"E
5		C034-Prasat Neang Khmao Temple, Srang, Kong Pisei	11°16'47.24"N, 104°36'25.50"E
6		C061-Kirirom National Park, Traeng Trayueng, Phnom Sruoch District	11°20'33.81"N, 104°02'9.77"E
7	Takeo	C036-Phnom Bayang, Kiri Vong District	10°38'28.1"N, 104°50'35.8"E
8		C037-Pha-aok Waterfall, Kiri Vong District	10°37'35.3"N, 104°51'30.2"E
9	Kampot	C041-Limestone mountain near To Tong, Dang Tong District	10°41'59.79"N, 104°31'30.14"E
10		C042-Prasat Phnom Totong, Banteay Meas District	10°41'49.6"N, 104°31'20.9"E
11		C043-Phnom Kampong Trach Cave Temple, Kampong Trach District	10°34'1.77"N, 104°28'6.13"E
12		C045-Phnom Kbal Romeas, Tuek Chhou District	10°37'0.08"N, 104°14'37.60"E
13		C046-Phnom Chhngok Cave, Tuek Chhou District	10°38'34.91"N, 104°16'4.07"E
14		C051-Sampov Pram Pagoda (site 1), Preah Monivong Bokor National Park	10°37'49.07"N, 104°01'3.12"E
15		C052-Popokvil Waterfall (site 2), Preah Monivong Bokor National Park	10°39'31.8"N, 104°03'03.2"E
16		C056-Prek Thnout Eco Park, Tuek Chhou District	10°36'44.29"N, 103°57'16.61"E
17	Kep	C047-Phnom Sorsia Temple, Ou Krasar, Krong Kaeb	10°33'53.57"N, 104°17'1.90"E
18		C048-Kep Beach, Prey Thom, Krong Kaeb	10°28'47.1"N, 104°17'32.8"E

All the specimens were identified to genus or species level based on shell characteristics by referring to the historical literature including original descriptions, recent catalogues of land snails from Laos by Inkhavilay et al. (2019), and the collections of the Muséum national ďHistoire naturelle (**MNHN**, Paris) and The Natural History Museum (**NHM**, London). The placement of each genus within higher order classification follows MolluscaBase (2020). All specimens were deposited at the Inland Fisheries Research and Development Institute (**IFReDI**) of the Fisheries Administration, Phnom Penh; Chulalongkorn University Museum of Zoology (**CUMZ**), Bangkok; Zoological Reference Collection of the Lee Kong Chian Natural History Museum, National University of Singapore (**ZRC**); The Natural History Museum, London (**NHMUK**).

### Study area

**Figure 2.**
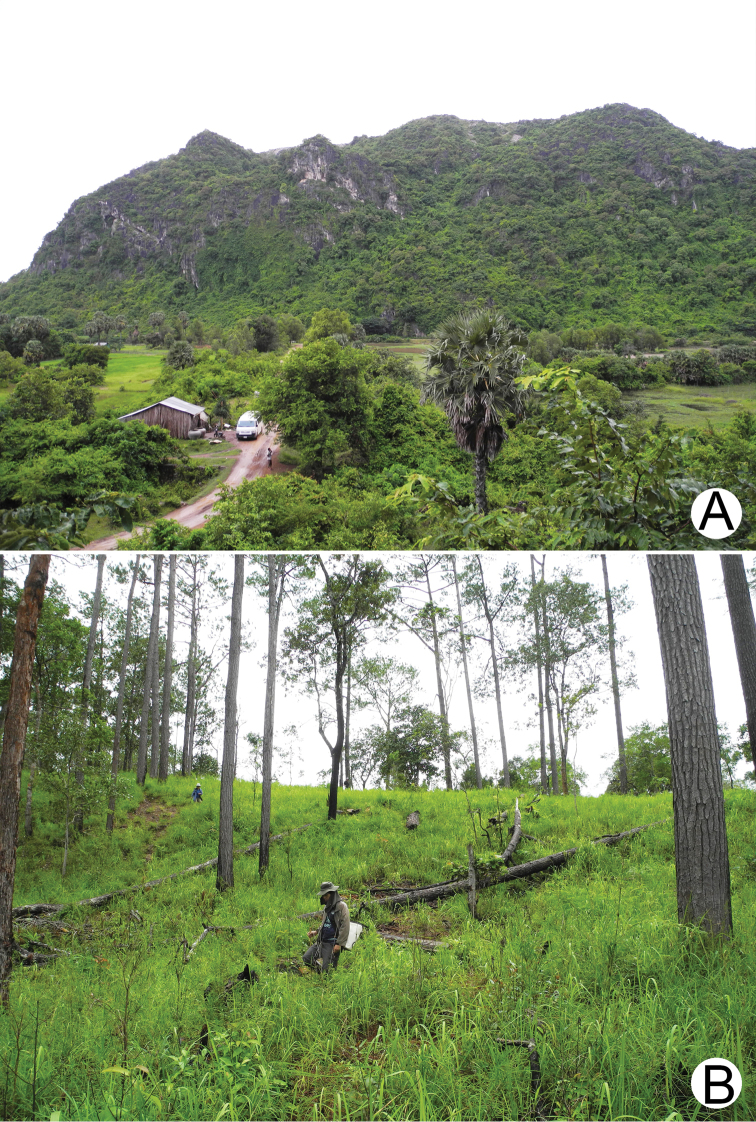
Collecting sites representing two main habitat types. **A** Limestone karsts with dry evergreen forest (September 2019; locality no. 10) **B** sandstones with grassland and coniferous forest (September 2019; locality no. 6).

Field surveys were conducted in karstic areas in southwestern Cambodia, Kampot Province. In addition, caves and cave-like chambers which provide appropriate microhabitats for karst-dwelling snails were also surveyed. This area has a monsoonal climate with wet season (May to November) and dry season (December to April). The karst landscape in Kampot is a small, isolated hill rising precipitously from the flat lowlands (Fig. 2A). The forest habitats surrounding the hill area have been degraded because of agricultural encroachment at the base of the hill and is enclosed by highly disturbed scrub vegetation, but the hill itself bears typical limestone vegetation on its cliff. Limestone quarrying is locally widespread.

Lowland habitats of the eastern areas of Kirirom National Park are a conglomerate of hills and a plateau reaching 900 m in elevation, straddling the Kampong Speu and Koh Kong Provinces. The bulk of the plateau is covered with a mosaic of grassland and a reticulated network of pine forest plantations (Fig. 2B). The slopes of the plateau support an evergreen forest interspersed with tracts of mixed deciduous and bamboo forests.

The Preah Monivong Bokor National Park, Kampot Province is in the southeastern portion of the Cardamom Mountain Ranges within a range known as the Elephant Mountains. The plateau reaches an elevation of 1,100 m, and the floral composition of this range is greatly affected by continuous, monsoonal winds arising from the Gulf of Thailand. The climate promotes a mixture of grassland and tropical moist forest that shrouds the upper elevations of the Bokor Plateau in thick fog for much of the year (Stuart and Emmett 2006, Grismer et al. 2008), the condition of which is also present during our time of surveys. The slopes of the area support tracts of primary evergreen forests and steep, fast flowing, rocky streams.

## Results

**Figure 3.**
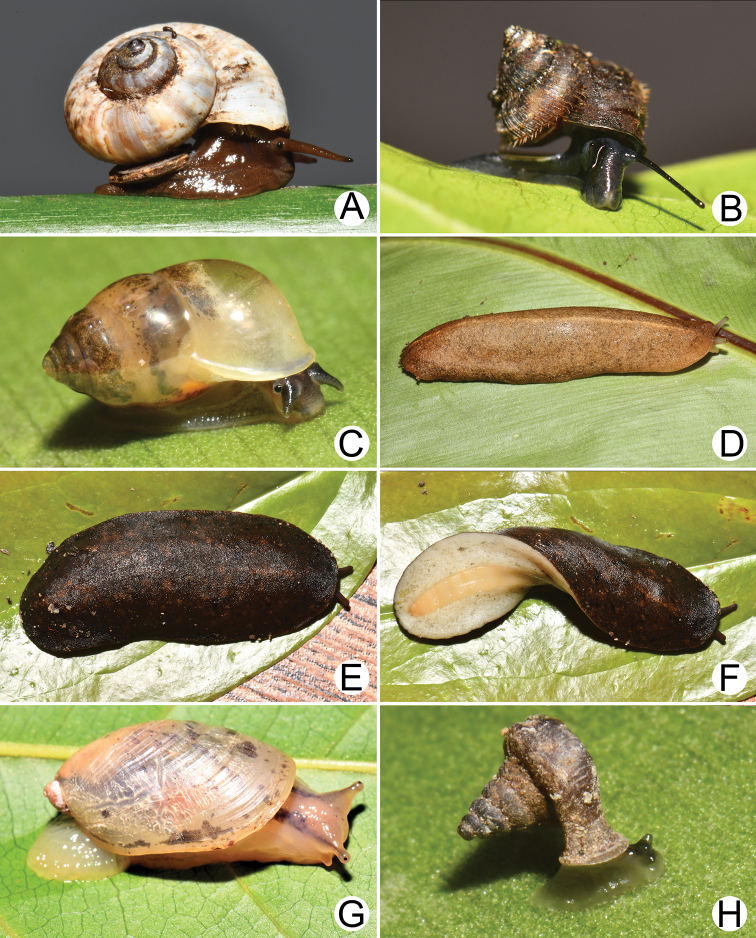
Living snails of **A***Lagocheilus klobukowskii* (Morlet, 1885) **B***Lagocheilus landesi* (Morlet, 1885) **C***Pupina crosseana* Morlet, 1883 **D***Valiguna siamensis* (Martens, 1867) **E, F***Valiguna* sp. **E** notum or dorsal view and **F** hyponotum or ventral view **G***Succinea tenuis* Morelet, 1865 and **H***Hypselostoma cambodjense* Benthem Jutting, 1962. All not to scale.

A total of 180 voucher specimen lots was collected over the survey. The total of 36 species (two Neritimorpha (Fig. 6A, B), six Caenogastropoda (Figs 3A–C, 6C–F, 7A–C), and 28 Heterobranchia fell within 25 genera and 13 families, including two non-native species (*Lissachatina fulica* and *Allopeas gracile*). The distribution data given under each species was retrieved from the past records.

### Subclass Neritimorpha

#### Family Hydrocenidae Troschel, 1847

*Georissa* **Blanford, 1864**

##### Georissa monterosatiana

Taxon classificationAnimaliaCycloneritidaHydrocenidae

Godwin-Austen & Neville, 1879

57959CF7-61E5-5AE9-AD45-9C02B68595E6

Fig. 6A

Georissa monterosatiana Godwin-Austen & Nevill, 1879: 739, 740, pl. 59, fig. 6. Type locality: Perak [Perak State, Malaysia]. Foon et al. 2017: 43, fig. 16c.

###### Material examined.

Locality no. 11: CUMZ-CM114 (10 shells; Fig. 6A). The snails were found to live on limestone wall syntopically with other *Hypselostoma* spp.

###### Distribution.

Thailand and Malaysia (Foon et al. 2017).

###### Remarks.

This species was described from “Perak” [Perak State, Malaysia]. The characters of this minute shell are elongate conic, yellowish to pale orange with darker colour on apex. The shell has four to five well-rounded whorls, with wide and impressed suture. Protoconch surface is smooth, with the following whorls sculptured with strong, regularly spaced spiral ribs and with ca. 9–11 spiral ribs on the last whorl (more thin spiral ribs at basal part of the last whorl). The aperture is round to slightly ovate, with a closed umbilicus. Although the specimens from Perak have denser spiral striation (Godwin-Austen and Nevill 1879, Foon et al. 2017), without a comprehensive revision of this genus the specimens from Cambodia were hereby identified as *G. monterosatiana*.

This species differs from *G. decora* Möllendorff, 1900 and *G. chrysacme* Möllendorff, 1900 both of which were described from “Touranne” [Da Nang, Vietnam], by having a conic shell with ca. ten strong spiral ribs on the last whorl. However, *G. decora* has an ovate conic shape with fine radial ribs on the last whorl, and *G. chrysacme* has an elongate conic shape with a deep and narrow suture. In addition, the shell shape of *G. monterosatiana* approaches the shape of *G. insulae*Khalik et al., 2019 from Borneo, but the former species has stronger and more undulated spiral ridges than the Bornean species (see Khalik et al. 2019).

##### Georissa carinata

Taxon classificationAnimaliaCycloneritidaHydrocenidae

Sutcharit & Jirapatrasilp sp. nov.

5B5771A6-B6EC-59E9-BA7E-E23BB4736AE5

http://zoobank.org/CA891381-B719-4A88-B4A9-B0E36BAB121E

Fig. 6B

###### Type material.

Holotype CUMZ-CM094/1 (Fig. 6B) from locality no. 11. Measurement: shell height 2.1 mm, shell width 1.5 mm and 4¼ whorls. Paratypes CUMZ-CM094/2 (9 shells) from locality no. 11; CUMZ-CM042 (21 shells), IFReDI (10 shells), ZRC (10 shells) and NHMUK (10 shells) from locality no. 9.

###### Type locality.

Phnom Kampong Trach Cave Temple, Kampong Trach District, Kampot Province, Cambodia, Locality no. 11 (10°34'1.77"N, 104°28'6.13"E).

###### Other material examined.

Locality no. 12: CUMZ-CM086 (23 shells). Locality no. 17: CUMZ-CM102 (18 shells).

###### Description.

Shell minute (shell height up to 2.1 mm, shell width up to 1.5 mm), conic, solid, translucent, yellowish to pale orange with darker colour on apex. Whorls 4¼, last whorl large ca. two-thirds of shell height. Protoconch ca. one whorl; sculpture nearly smooth and discontinuous spiral appearing immediately after protoconch. Following whorls slightly keeled, sculptured with thin and uneven growth lines; upper periphery with irregular and strong sculpture; below periphery with discontinuous spiral ribs. Sutures angular and impressed. Aperture round to slightly ovate. Umbilicus closed. Operculum unknown.

###### Etymology.

The Latin specific name *carinata* represents its keeled whorls of this new species.

###### Distribution.

This new species is found from Kampot and Kep Provinces. The snails were found to live on limestone wall syntopically with other *Hypselostoma* spp.

###### Remarks.

This new species differs from *G. bocourti* (Rochebrune, 1881) described from “Eaux douces de Preck-Scholl. Haut Mékong” [Chhloung District, Kratié Province, Cambodia], by having a conic shell with 4¼ whorls, which are slightly keeled and sculptured with thin and uneven growth lines without conspicuous spiral ribs. However, *G. bocourti* has a turriform shell with 6–7 whorls and sculptured with conspicuous spiral ribs (Rochebrune 1881a). *Georissa carinata* sp. nov. differs from *G. poirieri* Mabille, 1887 and *G. conspicua* Mabille, 1887 described from “Tonkin” [Vietnam] in that the latter two species are larger (shell height 3–5 mm, shell width 2½–3 mm) and has a turriform shell. In addition, *G. poirieri* has very thin, tight, wavy spiral ribs, while *G. conspicua* has uneven spiral ribs with additional protuberances unequally arranged along the longitudinal rows (Mabille 1887a, b).

### Subclass Caenogastropoda

#### Family Cyclophoridae Gray, 1847

***Cyclophorus* Montfort, 1810**

##### Cyclophorus amoenus

Taxon classificationAnimaliaArchitaenioglossaCyclophoridae

(Pfeiffer, 1854)

262B8A2B-3FD7-5E65-B8DF-40373B15A5F2

Fig. 6C

Cyclostoma (Cyclophorus) amoenum Pfeiffer, 1854[1852]: 62. Type locality: unknown.
Cyclophorus amoenus : Nantarat et al. 2014a: 4, 5, fig. 3a, b. Nantarat et al. 2014b: 103, table 1, fig. 1b. Do and Do 2019: 6, 7, table 1, figs 1.2, 13b, c.

###### Material examined.

Locality no. 10: CUMZ-CM053 (21 shells), CUMZ-CM054 (1 shell; Fig. 6C). Locality no. 7: CUMZ-CM0110 (1 shell). Locality no. 11: CUMZ-CM071 (2 shells). The empty shells were collected from the ground among leaf litter.

###### Distribution.

Cambodia and Thailand (Nantarat et al. 2014b). The distribution in Vietnam is doubtful (Do and Do 2019).

###### Remarks.

*Cyclophorus* is the genus encompassing highly variable shell morphology of both inter- and intraspecific entities. The demarcation among different species is poorly understood. Thus, both intensive and thorough revision and redescription require more effective taxonomic characters, e.g., morphometric analysis of large series of specimens and perhaps molecular phylogeny to clarify the exact species boundaries (see Nantarat et al. 2019).

This species was described from an unknown type locality. Later, the type specimens were examined and illustrated (Nantarat et al. 2014a) and then subsequently reported from central Thailand (Nantarat et al. 2014b). Do and Do (2019) reported this species from central and southern Vietnam, but it needs revision and confirmation by more studies. The distinguishing characters of this species are a less expanded apertural lip, thickened with multiple layers and with a highly variable colour pattern.

##### Cyclophorus paviei

Taxon classificationAnimaliaArchitaenioglossaCyclophoridae

Morlet, 1885

B28880AB-640C-5355-BF73-498D6C327782

Fig. 6D

Cyclophorus paviei Morlet, 1885[1884]: 389, 390, pl. 11, fig. 4, 4a. Type locality: Les montagnes de Dey-Crahom (terre rouge) [The mountains of Dey-Crahom (red earth)], sur la rive droite du grand fleuve [on the right bank of the great river (Mekong River)]. Do and Do 2019: 24, figs 9.39, 23c.
Cyclophorus (Eucyclophorus) paviei : Kobelt 1908: 615, 616, pl. 83, figs 7, 8.

###### Material examined.

Locality no. 13: CUMZ-CM119 (1 shell). Locality no. 6: CUMZ-CM176 (1 shell). Locality no. 9: CUMZ-CM036 (2 shells), CUMZ-C037 (1 shell; Fig. 6D), CUMZ-CM038 (1 specimen in ethanol). Locality no. 12: CUMZ-CM096 (1 shell). Locality no. 16: CUMZ-CM168 (2 shells), CUMZ-CM169 (1 specimen in ethanol), CUMZ-CM170 (1 specimen in ethanol). The snails were found to live on the ground among leaf litter.

**Figure 4.**
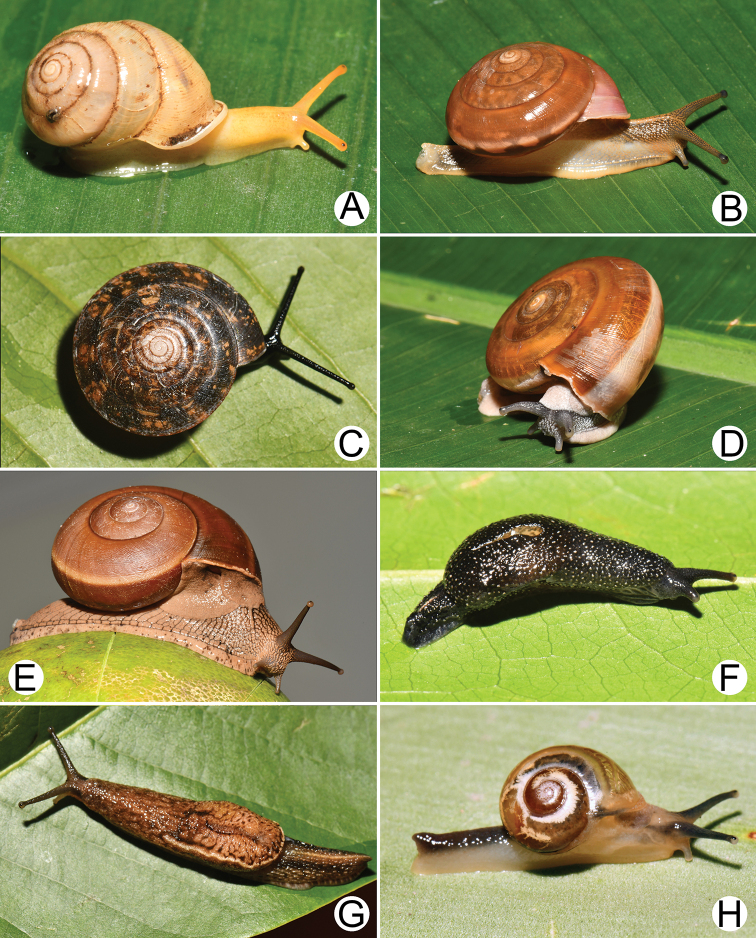
Living snails of **A***Haploptychius* sp. **B***Quantula weinkauffiana* (Crosse & Fischer, 1863) **C***Trochomorpha paviei* (Morlet, 1885) **D***Cryptozona siamensis* (Pfeiffer, 1856) **E***Hemiplecta distincta* (Pfeiffer, 1850) **F***Cambodiparmarion doroshenkoi* Kuznetsov & Kuzminykh, 1999 **G***Parmarion martensi* Simroth,^ 1893^ and **H***Durgella russeola* (Morelet, 1865). All not to scale.

###### Distribution.

Cambodia (Kobelt 1908). The distribution in Vietnam is doubtful (Do and Do 2019).

###### Remarks.

*Cyclophorus paviei* was described from “Les montagnes de Dey-Crahom”, from Cambodia. It differs from *C. cambodgensis* Morlet, 1885, which was described from the same area in having a smaller (shell width 32 mm) conical shell, with a white-yellowish apertural lip, while *C. cambodgensis* has a larger (shell width 42 mm) and turbinate conic shell, with an orange to reddish apertural lip.

#### *Opisthoporus* Benson in Pfeiffer, 1851

##### Opisthoporus bernardii

Taxon classificationAnimaliaArchitaenioglossaCyclophoridae

(Pfeiffer, 1862)

B24D7F7F-272C-5CAE-BE1A-4ADB522E60F7

Fig. 6E

Rhiostoma bernardii Pfeiffer, 1862: 45, 46, pl. 6, fig. 5. Type locality: Siam [Thailand]. Kobelt 1911: 761, pl. 111, figs 9, 10.
Cyclotus bernardii : Inkhavilay et al. 2019: 19, fig. 8d, e.
Opisthoporus bernardii : Do et al. 2020: 108, table 3.

###### Material examined.

Locality no. 9: CUMZ-CM043 (8 shells). Locality no. 13: CUMZ-CM127 (2 shells), CUMZ-CM118 (2 shells). Locality no. 10: CUMZ-CM062 (3 shells; Fig. 6E), CM063 (1 shell), CUMZ-CM064 (2 specimens in ethanol). The snails were found to live on the ground among leaf litter.

###### Distribution.

Cambodia, Laos and Thailand (Inkhavilay et al. 2019).

**Figure 5.**
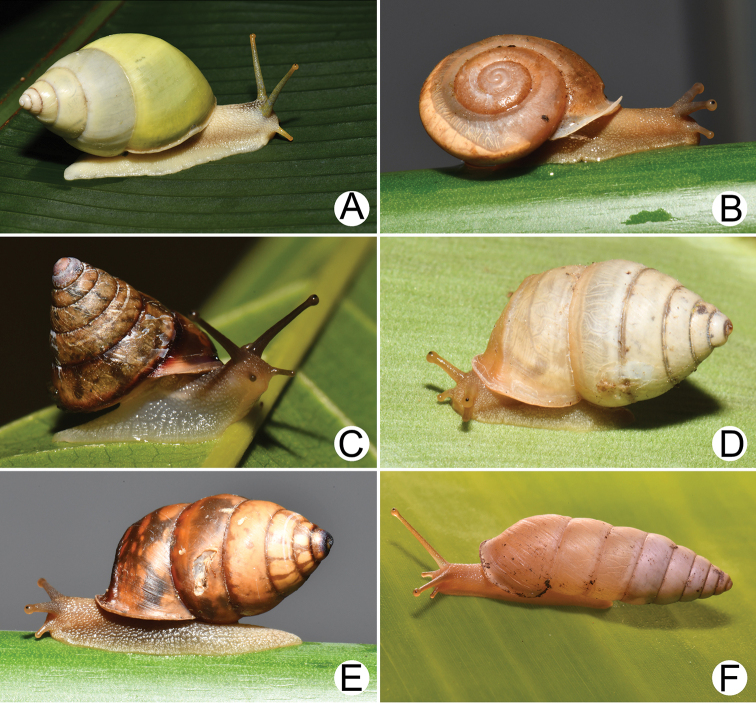
Living snails of **A***Amphidromus leucoxanthus* (Martens, 1864) **B***Trichochloritis norodomiana* (Morlet, 1883) **C***Ganesella perakensis* (Crosse, 1879) **D, E***Anceyoconcha rhombostoma* (Pfeiffer, 1861) **D** brownish morph and **E** dark brown morph and **F***Anceyoconcha chaudoensis* (Rochebrune, 1881) comb. nov. All not to scale.

###### Remarks.

*Opisthoporus bernardii* was originally described from “Siam” [Thailand], and it has been reported from Cambodia (Fischer 1973a) and Laos (Inkhavilay et al., 2019). The diagnostic characters of this species are the depressed helicoid shell, with thick or thin periostracum, circular aperture, and a short to long sutural tube (accessory breathing device) located just behind an apertural lip. Pfeiffer (1862) provided details of the operculum, which is calcareous and has a multi-spiral plate-like shape, while the operculum of *Rhiostoma* is thick calcareous and has a multi-spiral cup shape (Egorov 2009).

**Figure 6.**
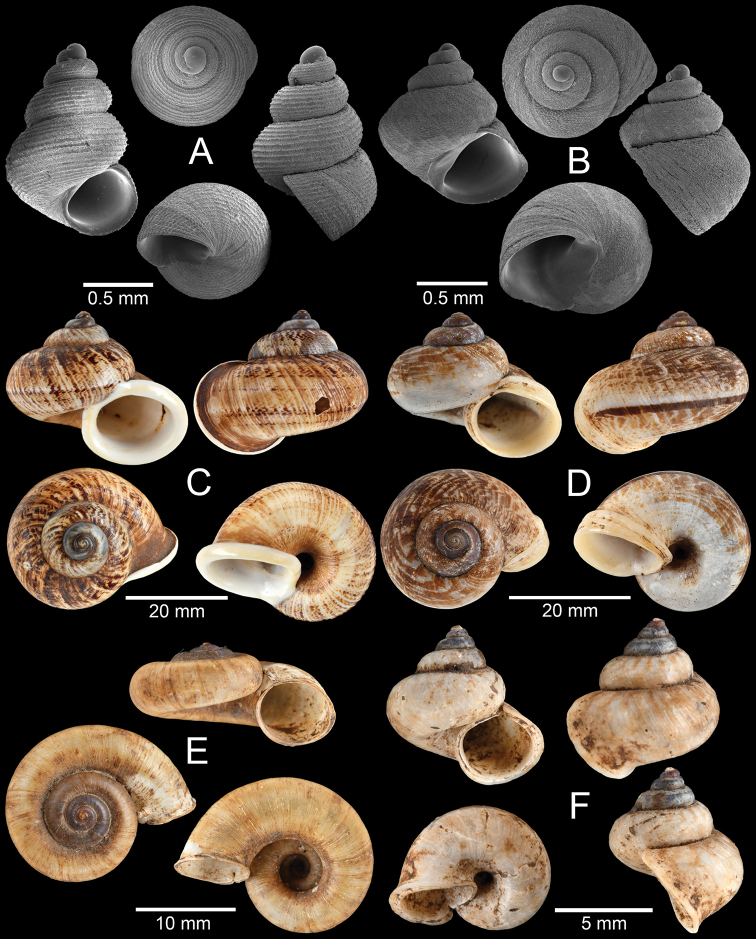
**A***Georissa monterosatiana* Godwin-Austen & Neville, 1879 **B***Georissa carinata* Sutcharit & Jirapatrasilp, sp. nov. **C***Cyclophorus amoenus* (Pfeiffer, 1854) **D***Cyclophorus paviei* Morlet, 1885 **E***Opisthoporus bernardii* (Pfeiffer, 1862) and **F***Lagocheilus landesi* (Morlet, 1885).

#### *Lagocheilus* Blanford, 1864

##### Lagocheilus klobukowskii

Taxon classificationAnimaliaArchitaenioglossaCyclophoridae

(Morlet, 1885)

A8BA2B42-822F-538E-889B-28DC9E7AA101

Figs 3A , 7A, B

Cyclophorus klobukowskii Morlet, 1885[1884]: 391, 392, pl. 12, fig. 1. Type locality: Near the Kamchay rapids, around the Kébal-Réméas cave (Kampot-Hatien road); commonly found on mountains, in forests, up to Compong-Som, and on the banks of Tap-Chéang. Fischer 1973a: 46, 47.
Lagocheilus klobukowskii : Inkhavilay et al. 2019: 19, 20, figs 9b, c, 18c.

###### Material examined.

**Figure 7.**
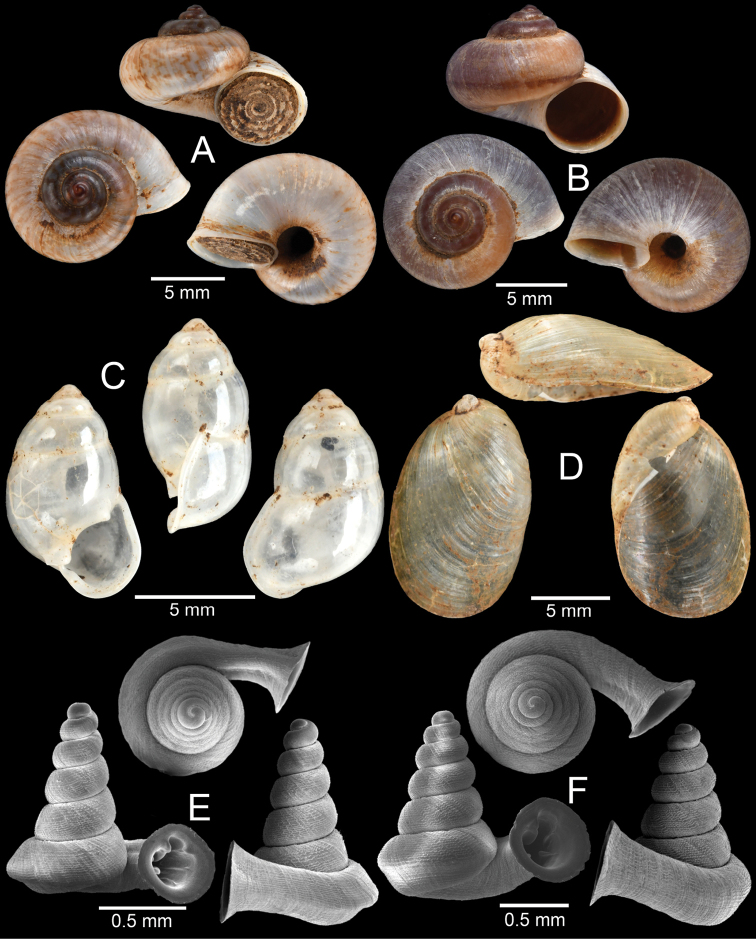
**A, B***Lagocheilus klobukowskii* (Morlet, 1885) **A** yellowish morph and **B** purplish-black morph **C***Pupina crosseana* Morlet, 1883 **D***Succinea tenuis* Morelet, 1865 and **E, F***Hypselostoma benetuitum* Vermeulen et al., 2019 **E** from locality no. 11 (type locality) and **F** from locality no. 17.

Locality no. 9: CUMZ-CM044 (7 shells), CUMZ-CM045 (12 specimens in ethanol). Locality no. 10: CUMZ-CM068 (3 specimens in ethanol). Locality no. 11: CUMZ-CM079 (2 shells). Locality no. 13: CUMZ-CM128 (3 shells), CM129 (53 specimens in ethanol; Fig. 3A). Locality no. 17: CUMZ-CM137 (18 shells; Fig. 7A, B). The snails were found to live on the ground among leaf litter and decaying wood, on tree trunks and limestone wall.

###### Distribution.

Cambodia and Laos (Inkhavilay et al. 2019).

###### Remarks.

This species was described from “…grotte de Kébal-Réméas (route de Kampot à Hatien) …”. We collected topotypic specimens that tend to have a variable shell colour from yellowish (Fig. 7A, see fig. 9b in Inkhavilay et al. 2019 for the syntype) to purplish-black (Fig. 7B). This limestone associated species has a wide distribution from southern Cambodia to eastern Laos (Inkhavilay et al. 2019). The snails are commonly found in montane forest, living on decaying wood, on tree trunks and exposed limestone.

*Lagocheilus klobukowskii* was originally placed in the genus *Cyclophorus* and later was transferred to the genus *Lagocheilus* (see Inkhavilay et al. 2019). The distinguishing characters from the genus *Cyclophorus* are a conic shell, an aperture thickened (not expanded), and a thick calcareous, multispiral and plate-like operculum, whereas *Cyclophorus* has a turbinate shell, a thick and expanded lip, and a corneous multispiral operculum.

##### Lagocheilus landesi

Taxon classificationAnimaliaArchitaenioglossaCyclophoridae

(Morlet, 1885)

614B5E69-1CA9-527D-9220-C0A030BE666E

Figs 3B , 6F

Cyclophorus landesi Morlet, 1885[1884]: 392, 393, pl. 11, fig. 5, 5a. Type locality: extrémité de la chaîne de ľÉléphant, non loin de la mer [Preah Monivong Bokor National Park, Kampot Province, Cambodia].
Cyclophorus laudesi [sic]: Fischer 1973a: 47.
Lagocheilus landesi : Inkhavilay et al. 2019: 20, fig. 9d.

###### Material examined.

Locality no. 11: CUMZ-CM080 (4 shells; Fig. 6F), CUMZ-CM082 (1 specimen in ethanol). Locality no. 12: CUMZ-CM104 (4 shells). Locality no. 14: CUMZ-CM152 (2 specimens in ethanol; Fig. 3B). The snails were found to live on tree trunks and leaves.

###### Distribution.

Cambodia and Laos (Inkhavilay et al. 2019).

###### Remarks.

This species was originally described from “Elephant Mountains” [Preah Monivong Bokor National Park, Kampot Province]. Both empty shells and living snails were collected from Preah Monivong Bokor National Park showing similar characteristics with the original description and illustration.

Living snails are typical of cyclophorids with a blackish soft body. The shell surface is furnished with thick and regular periostracal hairs. There are three rows of periostracal hairs on the upper periphery and several rows of short periostracal hairs below the periphery. The periostracum usually disappears in old and worn specimens. The species has a conic shell, an aperture thickened (not expanded), and the thick calcareous, multi-spiral and plate-like operculum characteristic of *Lagocheilus*.

#### Family Pupinidae Pfeiffer, 1853

***Pupina* Vignard, 1829**

##### Pupina crosseana

Taxon classificationAnimaliaArchitaenioglossaPupinidae

Morlet, 1883

283DD709-264C-5D8D-A991-BD4F39799F14

Figs 3C , 7C

Pupina crosseana Morlet, 1883: 108, 109, pl. 4, fig. 5. Type locality: Cambodge [Cambodia]. Fischer 1973a: 48.

###### Material examined.

Locality no. 7: CUMZ-CM029 (1 shell). Locality no. 9: CUMZ-CM039 (10 shells). Locality no. 10: CUMZ-CM066 (1 shell), CUMZ-CM067 (57 specimens in ethanol; Fig. 3C). Locality no. 11: CUMZ-CM072 (10 shells; Fig. 7C). Locality no. 12: CUMZ-CM097 (4 shells). Locality no. 13: CUMZ-CM133 (1 specimen in ethanol). Locality no. 17: CUMZ-CM142 (1 specimen in ethanol). The snails were found to live on the ground among leaf litter.

###### Distribution.

Cambodia (Fischer 1973a).

###### Remarks.

This species was originally described from “Cambodge” [Cambodia]. The diagnostic characters of this porcelain shell are a pupoid shell with varying shell colour from brownish to whitish, having a large, ovate last whorl ca. two-thirds of shell height. The shell has a thickened parietal callus, with a small posterior plica that is located some distance from an angular corner of aperture, which possesses a wide posterior canal. The anterior canal is a narrowly transverse slit overhung by a square and thickened columella plica. The aperture is circular with a white, thickened and slightly expanded lip.

### Subclass Heterobranchia

#### Family Veronicellidae Gray, 1840

***Valiguna* Grimpe & Hoffmann, 1925**

##### Valiguna siamensis

Taxon classificationAnimaliaSystellommatophoraVeronicellidae

(Martens, 1867)

59FA60F6-E456-561B-8B22-19E795F8A8D9

Fig. 3D

Vaginulus siamensis Martens, 1867: 68, pl. 5, fig. 3. Type locality: Petshaburi [Petchaburi Province, Thailand].
Valiguna siamensis : Inkhavilay et al. 2019: 48, figs 19b, 55b.

###### Material examined.

Locality no. 12: CUMZ-CM116 (8 specimens in ethanol; Fig. 3D). The slugs were found to live under leaf litter.

###### Distribution.

Laos, Sri Lanka and Thailand (Inkhavilay et al. 2019).

###### Remarks.

This species was recorded from several localities from this survey. They occur in anthropogenic habitats all over Laos and Thailand (Inkhavilay et al. 2019). This species has an elongate elliptical and flattened body, the notum with light brownish colour and with a pale yellow median stripe.

##### Valiguna

Taxon classificationAnimaliaSystellommatophoraVeronicellidae

sp.

33EC06F0-4B1E-5F82-B790-386CCBB85195

Fig. 3E, F

###### Material examined.

Locality no. 6: CUMZ-CM178 (1 specimen in ethanol; Fig. 3E, F). The slug was found to live under rotten logs.

###### Remarks.

This slug specimen was collected under rotten logs in grassland mixed with pine forest of Kirirom National Park at ca. an altitude of 660 m. They have a long elliptical and dorsolaterally flattened body. The dorsal side (notum) is thickened, with dark colour and scattered with brownish spots, and without median stripe. The ventral side (hyponotum) is with much lighter, pale creamy colouration, with tiny greyish spots distributed across hyponotum, and a narrow foot sole located in the middle. The foot sole is as long as and slightly narrower than the hyponotum, with pale yellowish brown colour. This slug is different from *V. siamensis* in having a blackish notum without the median stripe.

#### Family Succineidae Beck, 1837

***Succinea* Draparnaud, 1801**

##### Succinea tenuis

Taxon classificationAnimaliaStylommatophoraSuccineidae

Morelet, 1865

EA06A16E-E337-58B7-B934-EF322D27ECB2

Figs 3G , 7D

Succinea tenuis Morelet, 1865: 225, 226. Type locality: Cochinchina [South Vietnam]. Breure et al. 2018: 450, figs 1129, 1130.
Succinea tenella Morelet, 1875: 244, pl. 12, fig. 5 [unjustified emendation].

###### Material examined.

Locality no. 12: CUMZ-CM106 (2 shells), CUMZ-CM107 (2 specimens in ethanol; Figs 3G, 7D). The snails were found to live on tree trunks and leaves.

###### Distribution.

Cambodia, Thailand and Vietnam (Schileyko 2011).

###### Remarks.

We placed these Cambodian specimens under *S. tenuis* [= *S. tenella* Morelet, 1875] due to their appearance resembling the syntype that was recently figured in Breure et al. (2018: figs 1129, 1130). The diagnostic characters of this species are succiniform with thin and fragile shell, with ca. 3 whorls. The last whorl is very large, greatly expanded with approaching the shell height; the shell surface has strong irregular growth lines.

There is one species, *S. cochinchinensis* Crosse & Fischer, 1863 [= *S. cochinchinensis* Pfeiffer, 1865, junior homonym and junior synonym] reported from this area (Schileyko 2011). This nominal species was described from “Saigon”. However, the original description was very brief, and the type specimens have not been figured. Examination of types of *S. cochinchinensis* may reveal them to be conspecific with *S. tenuis*.

#### Family Vertiginidae Fitzinger, 1833

***Hypselostoma* Benson, 1856**

##### Hypselostoma benetuitum

Taxon classificationAnimaliaStylommatophoraGastrocoptidae

Vermeulen et al., 2019

516E782B-55AE-59A5-B6D5-7A569DCA142F

Fig. 7E, F

Hypselostoma benetuitum Vermeulen et al., 2019b: 32, 33, figs 64, 65. Type locality: Phnom Kampong Trach, Kampong Trach area, Kampot Province, Cambodia.

###### Material examined.

Locality no. 11: CUMZ-CM061 (6 shells; Fig. 7E). Locality no. 17: CUMZ-CM081 (19 shells; Fig. 7F). The snails were found to live on limestone wall syntopically with *Georissa* spp.

###### Distribution.

Kampong Trach area, Kampot Province, Cambodia (Vermeulen et al. 2019b).

###### Remarks.

The specimens from the type locality (locality no. 11; Fig. 7E) agree well with the drawing by Vermeulen et al. (2019b). This species tends to have a much smaller shell size and be less abundant than the syntopic congener *H. cambodjense* Benthem Jutting, 1962.

The specimens from locality no. 17 (Fig. 7F) could be identified to this species by having a smaller size than another congener, *H. cambodjense*. However, they differ slightly from the typical form in having less distinct peripheral ridges on the last whorl, while the protoconch, shell sculpture and major lamellae are identical to the topotype. Therefore, we consider these specimens to come within intraspecific variation.

##### Hypselostoma cambodjense

Taxon classificationAnimaliaStylommatophoraGastrocoptidae

Benthem Jutting, 1962

1F3031C6-4354-5EC3-80BD-088FAD95B40E

Figs 3H , 8A–C

Hypselostoma cambodjense Benthem Jutting, 1962: 3–5, fig. 1. Type locality: Phnom Can Long, à 6 km au Sud de Tuk Méas, Cambodge. Vermeulen et al., 2019b: 33.

###### Material examined.

**Figure 8.**
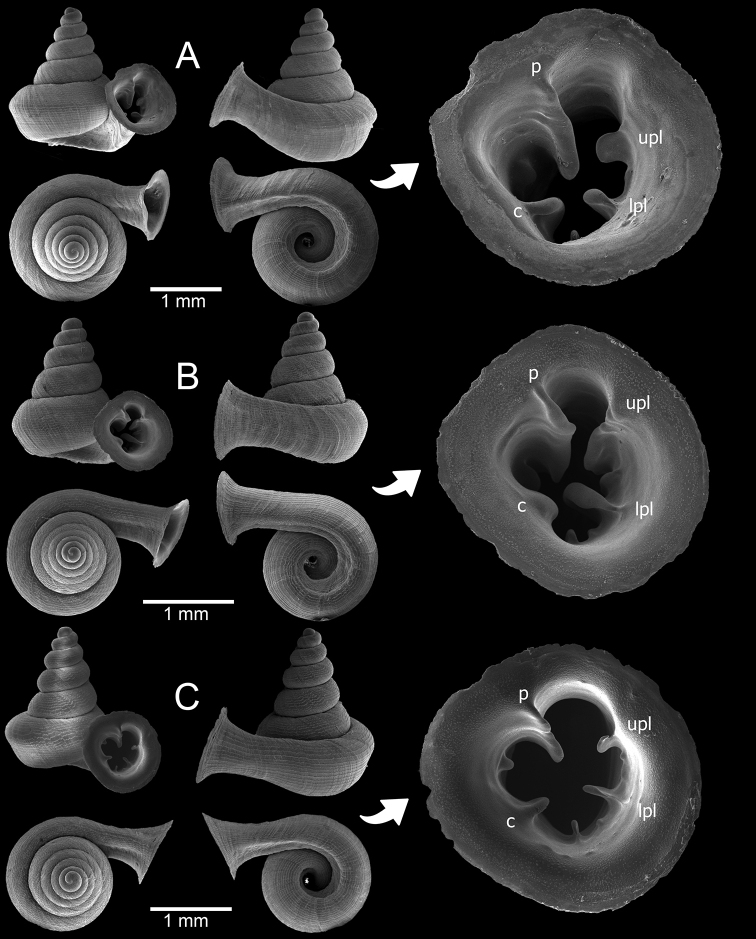
Morphological variation among *Hypselostoma cambodjense* Benthem Jutting, 1962 populations **A** from locality no. 9 (topotype) **B** from locality no. 11 and **C** from locality no. 17. The insets show the apertural dentition. Abbreviation: p, parietal lamella; upl, upper palatal lamella; lpl, lower palatal lamella; c, columellar lamella.

Locality no. 11: CUMZ-CM004 (77 shells; Fig. 8B). Locality no. 12: CUMZ-CM073 (63 shells). Locality no. 9: CUMZ-CM087 (122 shells; Figs 3H, 8A). Locality no. 17: CUMZ-CM138 (40 shells; Fig. 8C). The snails were found to live on limestone wall syntopically with *Georissa* spp.

###### Distribution.

Kampot Province, Cambodia and Ha Tien Town area, Kien Giang Province, Vietnam (Vermeulen et al. 2019b).

###### Remarks.

This species was originally described from limestone hills near “Tuk Méas”, probably in Banteay Meas area. The specimens collected from locality no. 9 are thus considered as topotypic specimens. These specimens agree well with the illustration in van Benthem Jutting (1962: fig. 1).

*Hypselostoma cambodjense* tends to be abundant and widely distributed in several karstic hills in southern Cambodia and Vietnam (see Vermeulen et al. 2019b). The populations from the localities no. 11 (Fig. 8B) and 17 (Fig. 8C) differ from the topotypic specimen (Fig. 8A) by having an obtusely angular to slightly shouldered last whorl, with the presence of minor lamellae, while the protoconch, shell sculpture, and major lamellae (parietal, upper palatal, lower palatal and columellar) are identical. Therefore, we treat them as a morphological variant of the same species.

#### Family Achatinidae Swainson, 1840

***Allopeas* Baker, 1935**

##### Allopeas gracile

Taxon classificationAnimaliaStylommatophoraAchatinidae

(Hutton, 1834)

B2EDA1FE-87E6-5097-B863-9DCB9ACC6E2F

Fig. 9A

Bulimus (?) gracilis (?) Hutton, 1834: 84, 85, 93. Type locality: Mirzapoor; Futtehpoor Sikra; between Agra and Neemuch [Uttar Pradesh and Madhya Pradesh States, India].
Allopeas gracilis [sic]: Inkhavilay et al. 2019: 50, fig. 21a–c.

###### Material examined.

**Figure 9.**
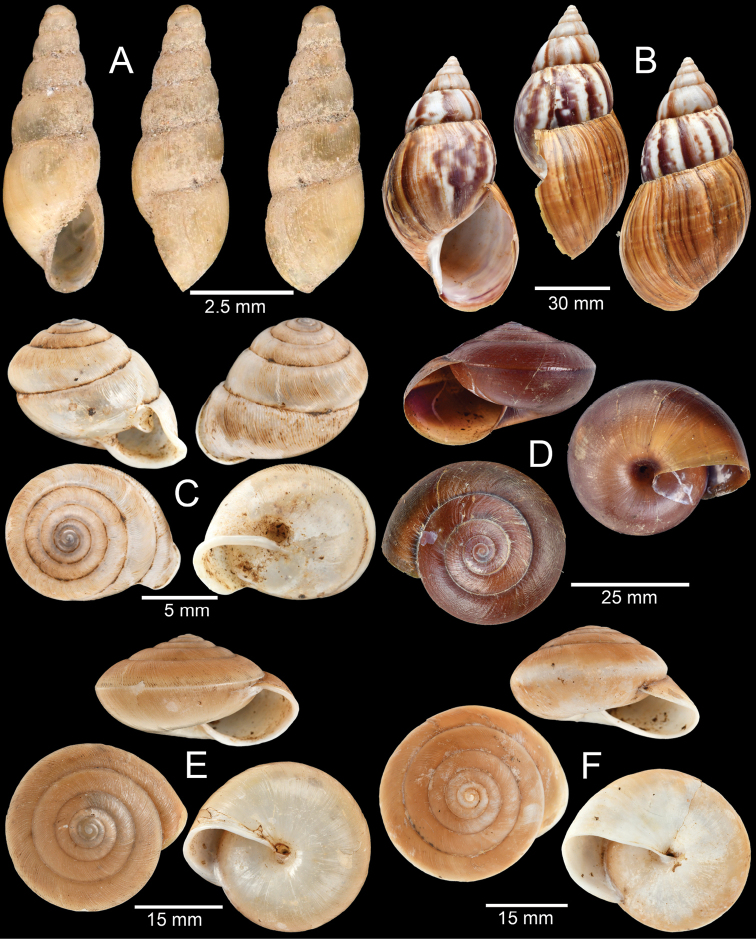
**A***Allopeas gracile* (Hutton, 1834) **B***Lissachatina fulica* (Bowdich, 1822) **C***Haploptychius* sp. **D***Dyakia* sp. and **E, F***Quantula weinkauffiana* (Crosse & Fischer, 1863) with **E** angular last whorl and **F** rounded last whorl.

Locality no. 12: CUMZ-CM105 (1 shell; Fig. 9A). Locality no. 6: CUMZ-CM177 (5 specimens in ethanol). The snails were found to live on the ground among leaf litter.

###### Distribution.

Pantropical and subtropical (Robinson et al. 2009).

###### Remarks.

This is the first official record of the non-native *A. gracile* in Cambodia. This species could be found in both natural and transformed anthropogenic habitats. This widespread and pantropical species has been introduced into many countries, including in greenhouses in temperate regions, and occurs throughout Laos, Thailand and Vietnam (Schileyko 2011, Inkhavilay et al. 2019).

#### *Lissachatina* Bequaert, 1950

##### Lissachatina fulica

Taxon classificationAnimaliaStylommatophoraAchatinidae

(Bowdich, 1822)

3E6BACC4-C635-59E3-824E-AE4521A0DFD0

Fig. 9B

Achatina fulica Bowdich, 1822: pl. 13, fig. 3. Type locality: unknown.
Lissachatina fulica : Inkhavilay et al. 2019: 49, fig. 20a.

###### Material examined.

Locality no. 10: CUMZ-CM065 (1 shell; Fig. 9B). Locality no. 12: CUMZ-CM095 (4 shells). The snails were found to live on tree trunks and on the ground among leaf litter.

###### Distribution.

Pantropical and subtropical (Fontanilla et al. 2014).

###### Remarks.

The likely origin of this species is from East Africa (Bequaert 1950). Currently, it has been introduced to many tropical countries, including all over Indochina (Fontanilla et al. 2014, Inkhavilay et al. 2019). The previous record of this species in Cambodia also indicated that this species is an intermediate host of the rat lungworm *Angiostrongylus cantonensis* (Brumpt et al. 1968).

#### Family Streptaxidae Gray, 1860

***Haploptychius* Möllendorff, 1905**

##### Haploptychius

Taxon classificationAnimaliaStylommatophoraStreptaxidae

sp.

48BD0608-0775-5246-BEA4-873C494E4369

Figs 4A , 9C

###### Material examined.

Locality no. 6: CUMZ-CM179 (1 specimen in ethanol). Locality no. 11: CUMZ-CM074 (12 shells; Fig. 9C), CUMZ-CM075 (1 shell), CUMZ-CM076 (14 specimens in ethanol; Fig. 4A). Locality no. 12: CUMZ-CM098 (8 shells), CUMZ-CM099 (1 shell), CUMZ-CM100 (9 specimens in ethanol). Locality no. 13: CUMZ-CM121 (21 shells), CUMZ-CM122 (1 specimen in ethanol). The snails were found to live on the ground among leaf litter in the limestone area.

###### Remarks.

This species is similar to *H. michaui* (Crosse & Fischer, 1863), but the latter is more ovate and less oblique in shell shape. In addition, this species can be distinguished from *H. pellucens* (Pfeiffer, 1863), *H. porrectus* (Pfeiffer, 1863) and *H. perlissus* Vermeulen et al., 2019 by having strong and prominent radial ridges. For comparison, the latter three species have a smooth to nearly smooth shell surface, *H. pellucens* has an oblique-ovate shell shape, *H. porrectus* and *H. perlissus* have an oblique heliciform shell shape (see Inkhavilay et al. 2016, and Vermeulen et al. 2019b for further comparison).

#### Family Dyakiidae Gude & Woodward, 1921

***Dyakia* Godwin-Austen, 1891**

##### Dyakia

Taxon classificationAnimaliaStylommatophoraDyakiidae

sp.

BFA9AF85-E3C1-5B10-942F-6394A0FEF337

Fig. 9D

###### Material examined.

Locality no. 15: CUMZ-CM155 (5 shells), CUMZ-CM156 (4 shells), CUMZ-CM157 (1 shell; Fig. 9D), CUMZ-CM158 (3 specimens in ethanol). The snails were found to live on tree trunks and leaves.

###### Remarks.

The large sinistral helicoid shell discriminates this species from most other species known in this region. This species can be distinguished from *Bertia cambojiensis* (Reeve, 1861) by having a brownish shell, with spirally undulated surfaces, while *B. cambojiensis* has a smooth surface (see Sutcharit et al. 2019b for further comparison).

The specimens from the Popokvil Waterfall (locality no. 15) located on the plateau of Preah Monivong Bokor National Park may be young individuals, as their shell size is relatively small compared to those of other congeners recorded from peninsular Thailand. It differs from *D. salangana* (Martens, 1883) and *D. retrorsa* (Gould, 1843) by having a dark brown shell, with wide angle of peripheral keels. In contrast, *D. retrorsa* tends to have sharp peripheral keel, *D. salangana* has round periphery and usually with brownish peripheral band, and both species are pale brownish in shell colour (BEDO 2017).

#### *Quantula* Baker, 1941

##### Quantula weinkauffiana

Taxon classificationAnimaliaStylommatophoraDyakiidae

(Crosse & Fischer, 1863)

9963D788-2D1B-5D3C-883F-9E6D421B1761

Figs 4B , 9E, F

Helix weinkauffiana Crosse & Fischer, 1863: 350, 351. Type locality: Cochinchine [Southern Vietnam].
Quantula weinkauffiana : Inkhavilay et al. 2019: 71, figs 32b–d, 55h.

###### Material examined.

Locality no. 1: CUMZ-CM002 (8 shells). Locality no. 2: CUMZ-CM006 (10 shells). Locality no. 5: CUMZ-CM011 (2 shells). Locality no. 7: CUMZ-CM013 (82 shells), CUMZ-CM014 (1 shell), CUMZ-CM015 (1 shell + 1 specimen in ethanol; Fig. 4B). Locality no. 9: CUMZ-CM034 (7 shells), CUMZ-CM035 (3 shells). Locality no. 10 CUMZ-CM052 (1 shell). Locality no. 12: CUMZ-CM093 (5 shells). Locality no. 13: CUMZ-CM120 (5 shells). Locality no. 17: CUMZ-CM135 (3 shells). Locality no. 18: CUMZ-CM143 (3 shells), CUMZ-CM144 (1 shell). Locality no. 16: CUMZ-CM166 (4 shells), CUMZ-CM177 (1 shell). Locality no. 6: CUMZ-CM174 (9 shells), CUMZ-CM175 (2 shells; Fig. 9E, F). The small juveniles were found on tree trunks and leaves, while the adults were found to live on the ground among leaf litter.

###### Distribution.

Cambodia, Laos, Thailand and Vietnam (Schileyko 2011, Inkhavilay et al. 2019).

###### Remarks.

This species was originally described from “Cochinchina”. The distinguishing characters are a depressed-conic to conic shell shape and brownish shell colour. The last whorl is round to angular, with upper shell surface sculptured with fine radial ridges, below the periphery the surface is usually smooth. The aperture is sub-circular, with lip thickened in adult specimens. However, this species tends to have a highly variable shell from depressed-conic to dome-shaped shell, and the last whorl rounded (Fig. 9F) to angular (Fig. 9E).

The living snail has reticulated skin, yellowish to pale orange body, usually with dark longitudinal anterior stripes. *Quantula weinkauffiana* is considered to be a common species in Cambodia, where they can be found in both natural and highly disturbed human-modified habitats, such as agricultural plantations. Although Brumpt et al. (1968) reported that *Q. striata* from Cambodia is an intermediate host of the rat lungworm *Angiostrongylus cantonensis*, the land snail species in that study was more likely *Q. weinkauffiana*.

#### Family Trochomorphidae Möllendorff, 1890

***Trochomorpha* Albers, 1850**

##### Trochomorpha paviei

Taxon classificationAnimaliaStylommatophoraTrochomorphidae

(Morlet, 1885)

EDC62306-0411-5BDB-93D2-C99EEEB8A0C5

Figs 4C , 10A

Helix paviei Morlet, 1885[1884]: 386, 387, pl. 11, fig. 1, 1a. Type locality: dans les forêts, entre Kampot et Phnom-Penh, particulièrement près des rapides de Kamchay (rivière de Kampot), sur les bois pourris et les petite plantes [In forests, between Kampot and Phnom Penh, especially near the rapids Kamchay (Kampot River), on rotten wood and small plants].
Trochomorpha paviei : Inkhavilay et al. 2019: 72, figs 33a, b, 56a.

###### Material examined.

**Figure 10.**
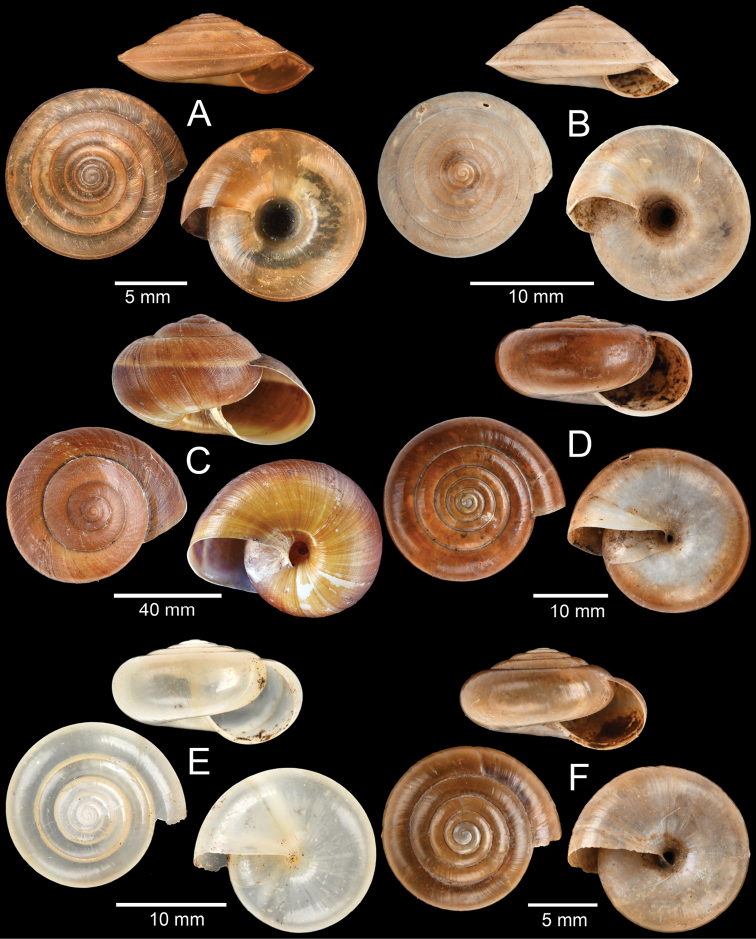
**A***Trochomorpha paviei* (Morlet, 1885) **B***Trochomorpha* sp. **C***Hemiplecta distincta* (Pfeiffer, 1850) **D***Sarika* sp. 1 **E***Sarika* sp. 2 and **F***Sarika* sp. 3.

Locality no. 14: CUMZ-CM153 (3 specimens in ethanol). Locality no. 15: CMZ-CM162 (2 shells), CUMZ-CM163 (14 specimens in ethanol; Figs 4C, 10A), CUMZ-CM164 (6 specimens in ethanol). The snails were found to live on tree trunks and on the ground among leaf litter.

###### Distribution.

Cambodia, Laos and Vietnam (Schileyko 2011, Inkhavilay et al. 2019).

###### Remarks.

This species was originally described from “Dans les forêts, entre Kampot et Phnom-Penh”. The unique characters are a depressed conic shell (shell width 12 mm) with a very strong and sharp peripheral keel, and a widely opened and deep umbilicus. The shell surface has thin and regular radial ridges, and very thin spiral ridges. Based on shell morphology, *T. paviei* closely resembles *T. saigonensis* (Crosse, 1867) that was described from “Poulo-Condor and Saigon, Cochinchine”. The latter species is slightly smaller (shell width 11 mm), having the last whorl with a wide angled peripheral keel and being slightly convex below the periphery. The type specimens of both species were recently figured in Inkhavilay et al. (2019: fig. 33a, c). However, we hesitate to lump them together, as additional information is necessary to further confirm their status.

##### Trochomorpha

Taxon classificationAnimaliaStylommatophoraTrochomorphidae

sp.

4907C676-3C20-5516-A416-2FE97F57D66C

Fig. 10B

###### Material examined.

Locality no. 10: CUMZ-CM057 (2 shells; Fig. 10B), CUMZ-CM058 (1 shell), CUMZ-CM059 (1 specimen in ethanol). Locality no. 13: CUMZ-CM134 (3 shells). The snail was found to live on a tree trunk.

###### Remarks.

The specimens from Prasat Phnom Totong (locality no. 10) have a conic shell with a very strong and sharp peripheral keel, widely opened and deep umbilicus, and slightly convex below the periphery. The shell surface has irregular growth lines and very thin spiral ridges. These specimens tend to differ from *T. paviei* and *T. saigonensis* in having a larger shell (shell width 14 mm), an elevated domed spire, more whorls, and being nearly flat below the periphery. However, the identification is provisional, and further evidence from examination of genitalia or DNA will be necessary to elucidate their status.

#### Family Ariophantidae Godwin-Austen, 1883

***Cryptozona* Mörch, 1872**

##### Cryptozona siamensis

Taxon classificationAnimaliaStylommatophoraAriophantidae

(Pfeiffer, 1856)

C459181B-410C-5886-80E0-0BA6909196F3

Fig. 4D

Helix siamensis Pfeiffer, 1856: 32. Type locality: Siam [Thailand].
Hemiplecta dichromatica Morlet, 1889: 124, 175, 176, pl. 6, fig. 2. Type locality: de Srakéo à Ang-Son (Siam) [Srakeo Province, Thailand].
Cryptozona siamensis : Inkhavilay et al. 2019: 75, 76, figs 35a, 56b.

###### Material examined.

Locality no. 4: CUMZ-CM147 (1 specimen in ethanol; Fig. 4D). Locality no. 18: CUMZ-CM146 (2 shells). The snails were found to live on the ground among leaf litter.

###### Distribution.

Cambodia, Laos, Malaysia, Singapore and Thailand (Tan et al. 2016, Inkhavilay et al. 2019).

###### Remarks.

This widespread species has recently been recorded from Singapore and Peninsular Malaysia (Tan et al. 2016), Laos (Inkhavilay et al. 2019) and Yunnan, China (C. Sutcharit, pers. obs.). In Thailand, *C. siamensis* is found throughout the country and the allozyme analysis by Prasankok and Panha (2011) indicates a surprisingly high level of genetic homogeneity among populations. This suggests that *C. siamensis* probably occupies almost all habitat types through accidental introduction or horticultural trade activities, and this species is especially abundant in human-modified landscapes.

The historical record of this species from Cambodia was probably under the name “*Hemiplecta dichromatica* Morlet, 1889” which was subsequently considered to be conspecific with this species (Fischer and Dautzenberg 1904). In this survey, *C. siamensis* was found from a suburb of Krong Chhbar Mon (locality no. 4), in which its habitats are associated with human activities. In contrast, *Q. weinkauffiana* could be found commonly in both natural forest and anthropogenic habitats.

#### *Hemiplecta* Albers, 1850

##### Hemiplecta distincta

Taxon classificationAnimaliaStylommatophoraAriophantidae

(Pfeiffer, 1850)

4A498BFE-3341-5F92-8207-B3B3E08EF2DA

Figs 4E , 10C

Helix distincta Pfeiffer, 1850: 69, 70. Type locality: insulis Moluccis [Molucca Islands].
Hemiplecta distincta : Inkhavilay et al. 2019: 76, figs 35b, c, 56c.

###### Material examined.

Locality no. 7: CUMZ-CM021 (6 shells), CUMZ-CM022 (1 specimen in ethanol), CUMZ-CM023 (1 specimen in ethanol). Locality no. 11: CUMZ-CM069 (2 shells), CUMZ-CM070 (1 specimen in ethanol; Fig. 4E). Locality no. 12: CUMZ-CM115 (1 shell). Locality no. 3: CUMZ-CM123 (1 specimen in ethanol). Locality no. 17: CUMZ-CM136 (4 shells). Locality no. 18: CUMZ-CM145 (1 shell). Locality no. 14: CUMZ-CM149 (1 shell). Locality no. 16: CUMZ-CM171 (1 shell; Fig. 10C), CUMZ-CM172 (1 specimen in ethanol). Locality no. 6: CUMZ-CM180 (3 specimens in ethanol). The small juveniles were found on tree trunks and leaves, while the adults were found to live on the ground among leaf litter.

###### Distribution.

Cambodia, Laos, Thailand and Vietnam (Schileyko 2011, Inkhavilay et al. 2019).

###### Remarks.

This is one of the largest land snail species recorded from Indochina. *Hemiplecta distincta* has a wide distribution from Southern Vietnam, throughout Cambodia, northeastern Thailand, and throughout Laos (Schileyko 2011, Inkhavilay et al. 2019). The snails are widely used as local food and are gathered for personal consumption or sold in high quantities, especially in Northeastern Thailand and Laos (Panha 1987). This species is an intermediate host of the rat lungworm *Angiostrongylus cantonensis* in Cambodia (Brumpt et al. 1968).

#### *Sarika* Godwin-Austen, 1907

##### Sarika

Taxon classificationAnimaliaStylommatophoraAriophantidae

sp. 1

E739D1F4-B036-5F9A-9DD1-1A85F87D43A8

Fig. 10D

###### Material examined.

Locality no. 9: CUMZ-CM032 (12 shells), CUMZ-CM033 (3 shells). Locality no. 10: CUMZ-CM050 (24 shells), CUMZ-CM051 (1 shell; Fig. 10D). The empty shells were collected among leaf litter in the limestone area.

###### Remarks.

The common ground dwelling snail genus *Sarika* is probably restricted to the Indochina region (Godwin-Austen 1907). Identifications at species level in *Sarika* based solely on shells cannot be achieved with confidence because of the limited distinguishing shell characters. Species level distinguishing characters in *Sarika* are based mainly on their reproductive anatomy.

However, this specimen can be discriminated from *S. bocourti* (Morelet, 1875) by having a reddish-brown shell with a wide whitish or creamy area surrounding the umbilicus. *Sarika bocourti*, which is described from “Battambang, Cambodje”, has a uniform brownish shell (see Breure et al. (2018: fig. 135) for the syntype).

##### Sarika

Taxon classificationAnimaliaStylommatophoraAriophantidae

sp. 2

65BA7FFA-3277-5FA6-8281-5689E7EEFB87

Fig. 10E

###### Material examined.

Locality no. 12: CUMZ-CM089 (4 shells). Locality no. 13: CUMZ-CM117 (2 shells; Fig. 10E). The empty shells were collected among leaf litter in the limestone area.

###### Remarks.

Recently, Vermeulen et al. (2019b) introduced a species *Macrochlamys psyche* Vermeulen et al., 2019 based on the shell alone. However, its generic placement remains uncertain since genitalia data is still lacking. Godwin-Austen (1907) and Pholyotha et al. (2018) stated that most of the species attributed to “*Macrochlamys*” in Southeast Asia belong to the genus *Sarika*.

This species is distinguished from the other known *Sarika* species in Cambodia by having a milky-coloured shell, flattened spire, and a relatively large shell (largest shell diameter 25 mm). These specimens differ from *M. psyche* in having a nearly flattened to slightly elevated spire, with a slightly shouldered last whorl and milky shell colour, while *M. psyche* has a slightly sunken spire, with a well-rounded last whorl and whitish shell colour (see Vermeulen et al. (2019b) for comparison).

##### Sarika

Taxon classificationAnimaliaStylommatophoraAriophantidae

sp. 3

CE4D967F-8241-5634-A485-A3EBD78E6A4F

Fig. 10F

###### Material examined.

Locality no. 11: CUMZ-CM088 (10 shells), CUMZ-CM092 (1 shell; Fig. 10F). The empty shells were collected among leaf litter in the limestone area.

###### Remarks.

The specimens from Phnom Kbal Romeas (locality no. 12) have a small shell (diameter ca. 10 mm), which is depressed, slightly thick, translucent, shiny, and pale reddish-brown. The shell surface is smooth with obvious irregular growth lines. The shell has 5 to 6 whorls, with wide and shallow suture. The spire is convex, with an elevated apex. The last whorl has a well-rounded periphery, with an ovate-lunate aperture and a simple lip. An umbilicus is widely open and deep.

These specimens can be distinguished from *Macrochlamys psyche*, *Sarika* sp. 1 and sp. 2 by having a small size and slightly elevated spire. In contrast, *M. psyche* and *Sarika* sp. 1 have a large, whitish shell and a flatten to slightly shrunken spire, while *Sarika* sp. 2 has a larger, reddish-brown shell with whitish area surrounding the umbilicus. Live specimens are required so that the anatomical characters can be used to discriminate among the species.

#### Family Helicarionidae Bourguignat, 1877

***Cambodiparmarion* Kuznetsov & Kuzminykh, 1999**

##### Cambodiparmarion doroshenkoi

Taxon classificationAnimaliaStylommatophoraAriophantidae

Kuznetsov & Kuzminykh, 1999

7F1D24E2-D479-5F7E-96A0-A98EDACC1FA6

Figs 4F , 11A

Cambodiparmarion doroshenkoi Kuznetsov & Kuzminykh, 1999: 113–116, figs 1, 2. Type locality: In tropical forest between Motel Lomherkay and Hotel Koh Pos, SW end of Kompong Som [= Sihanoukville], Kompong Som district, Kampot province, Cambodia.

###### Material examined.

**Figure 11.**
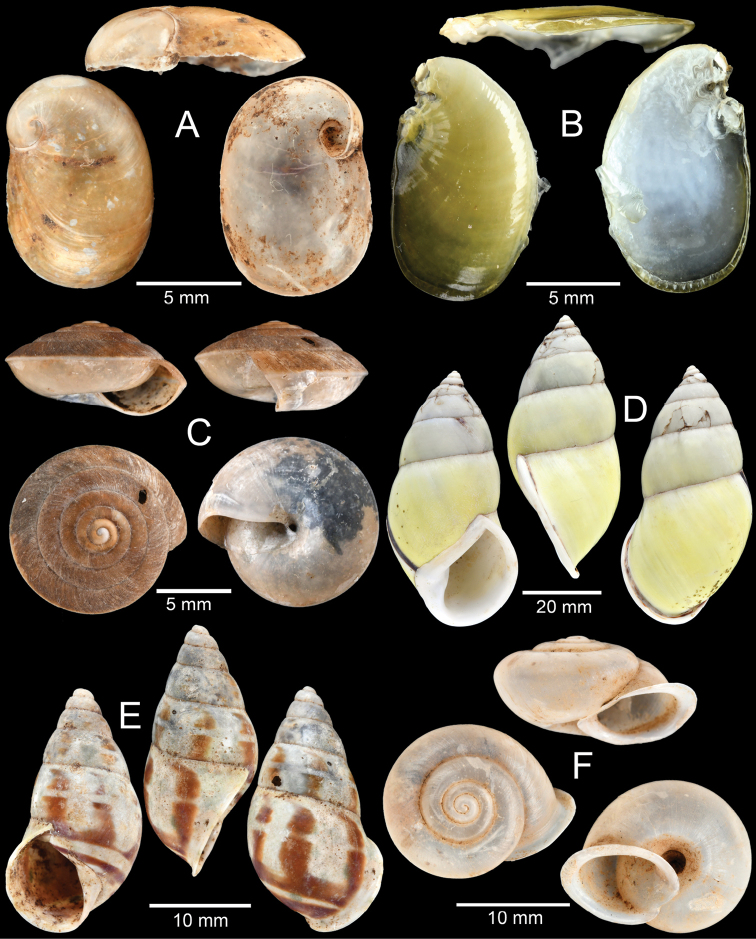
**A***Cambodiparmarion doroshenkoi* Kuznetsov & Kuzminykh, 1999 **B***Parmarion martensi* Simroth,^ 1893^**C***Sesara* sp. **D***Amphidromus leucoxanthus* (Martens, 1864) **E***Amphidromus semitessellatus* (Morlet, 1885) and **F***Trichochloritis norodomiana* (Morlet, 1883).

Locality no. 12: CUMZ-CM108 (4 shells). Locality no. 13: CUMZ-CM130 (2 shells), CUMZ-CM131 (1 specimen in ethanol; Fig. 4F). Locality no. 11: CUMZ-CM083 (1 shell; Fig. 11A), CUMZ-CM084 (9 shells). The semi-slug was found to live on tree trunks and leaves in the limestone area.

###### Distribution.

Known only from the type locality (Kuznetsov and Kuzminykh 1999).

###### Remarks.

This monotypic genus was recently described. It differs from the genus *Microparmarion* Simroth,^ 1893^ in having an enlarged and long cylindrical gametolytic sac, while the latter has a short and globular gametolytic sac. When *C. doroshenkoi* was described, the authors did not mention the characters used to discriminate this species from *Parmarion martensi* Simroth,^ 1893^. Here, we provide supplementary distinguishing characters as *C. doroshenkoi* has a solid, ear-shape shell with ca. 2 whorls, a blackish body and mantle, and a long flagellum, while *P. martensi* has a thin nail-shape shell with a trace of shell coiling, a greyish to blackish body and a short flagellum (Simroth 1893, Kuznetsov and Kuzminykh 1999).

Breure et al. (2018: figs 1196, 1197) illustrated the syntype of *Vitrina unguiculus* Morelet, 1865 described from “Cochinchina”. The syntypes are very similar in all characters to the shells of *C. doroshenkoi* examined herein. Further collections are needed to generate anatomical and molecular data to confirm whether they are conspecific or not.

#### *Parmarion* Fischer, 1855

##### Parmarion martensi

Taxon classificationAnimaliaStylommatophoraAriophantidae

Simroth, 1893

DC8AC934-7129-564A-8132-4385BDFBAB51

Figs 4G , 11B

Parmarion martensi Simroth,^ 1893^: 107, 108, pl. 7, fig. 8, pl. 8, figs 20–22. Type locality: Cambodja [Cambodia]. Inkhavilay et al. 2019: 81, figs 19g, 57d.

###### Material examined.

Locality no. 14: CUMZ-CM150 (1 shell), CUMZ-CM151 (6 specimens in ethanol; Figs 4G, 11B). Locality no. 15: CUMZ-CM165 (3 specimens in ethanol). The semi-slugs were found to live on tree trunks and leaves.

###### Distribution.

Cambodia, Laos, Malaysia and Singapore (Maassen 2001, Inkhavilay et al. 2019).

###### Remarks.

This semi-slug bears a small shell (plate or nail-like without a trace of shell coiling), in which the shell is usually entirely covered with movable mantle lobes. *Parmarion martensi* has also been reported as an introduced species to Samoa and Hawaii (Cowie et al. 2018).

#### *Durgella* Blanford, 1863

##### Durgella russeola

Taxon classificationAnimaliaStylommatophoraHelicarionidae

(Morelet, 1865)

D96F0748-53E1-5B2B-845F-79B4D0954B75

Fig. 4H

Vitrina russeola Morelet, 1865: 225. Type locality: Cochinchina. Breure et al. 2018: 416, figs 980, 981.
Megaustenia russeola : Schileyko 2011: 32.

###### Material examined.

Locality no. 7: CUMZ-CM030 (3 specimens in ethanol; Fig. 4H). The snails were found to live on tree trunks and leaves.

###### Distribution.

Vietnam (Schileyko 2011).

###### Remarks.

A syntype of *Durgella russeola* (Morelet, 1865) was figured in Breure et al. (2018: figs 980, 981); the type locality is “Cochinchina”. The shell of this species is globose, very thin (leather like or membranous) with a few calcareous elements, transparent, ovate conic. The shell has approximately 3 to 4 whorls, with an expanded last whorl, ovate aperture and closed umbilicus. Although there is no anatomical information at the moment, the distinct shell characteristics suggest that this species belongs to the semi-slug genus *Durgella*. Further additional anatomical examination is necessary since the shell morphology is insufficient for species identification.

#### *Sesara* Albers, 1860

##### Sesara

Taxon classificationAnimaliaStylommatophoraAriophantidae

sp.

456EF9A1-F892-5A09-BB2F-C813B4D198A7

Fig. 11C

###### Material examined.

Locality no. 7: CUMZ-CM031 (1 shell; Fig. 11C). The empty shell was collected among leaf litter in the limestone area.

###### Remarks.

The single shell was collected from Phnom Bayang (locality no. 7). It is distinguished from *Sesara polita* Vermeulen et al., 2019 and *S. sesarella* Vermeulen et al., 2019, which were recently described from Kampot Province, Cambodia, in having strong and prominent radial ridges continuously covering the entire teleconch and last whorl, and without any apertural lamella. *Sesara polita* possesses a smooth shell surface usually with one small basal and one transverse palato-basal lamellae, while *S. sesarella* has strong radial ridges on the teleconch, with a smooth last whorl, and has one thick and transverse parietal, one small palato-basal lamella and one small palatal lamella (Vermeulen et al. 2019b).

#### Family Camaenidae Pilsbry, 1895

***Amphidromus* Albers, 1850**

##### Amphidromus leucoxanthus

Taxon classificationAnimaliaStylommatophoraCamaenidae

(Martens, 1864)

4392F96C-9762-5E88-9AAE-9603DB4DEBB0

Figs 5A , 11D

**Figure 12.**
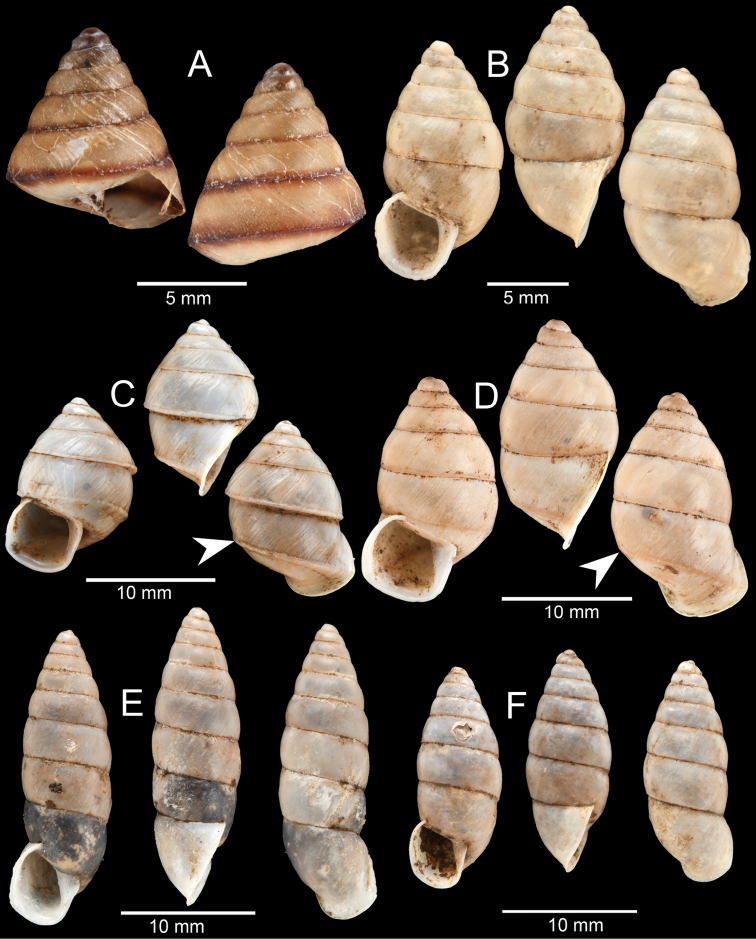
**A***Ganesella perakensis* (Crosse, 1879) **B***Anceyoconcha siamensis obesula* (Ancey, 1888) comb. nov. **C, D***Anceyoconcha rhombostoma* (Pfeiffer, 1861). A white arrowhead indicates strong peripheral keel on the last whorl, the distinguished character of this species and **E, F***Anceyoconcha chaudoensis* (Rochebrune, 1881) comb. nov.

Bulimus leucoxanthus Martens, 1864: 526. Type locality: unknown.
Amphidromus (Amphidromus) atricallosus leucoxanthus : Sutcharit and Panha 2006: 20, 21, figs 3p, 4a–e, 10d–f, 12b, 14c, d, 15c.

###### Material examined.

Locality no. 7: CUMZ-CM018 (2 shells; Fig. 11D), CUMZ-CM020 (2 specimens in ethanol; Fig. 5A) . The snails were found to live on tree trunks and leaves.

###### Distribution.

Eastern Thailand (Sutcharit and Panha 2006).

###### Remarks.

This species has been formerly treated as a subspecies within *A. atricallosus* (Sutcharit and Panha 2006). However, this species differs from *A. atricallosus* by having an elongate conic shell with thick or thin and whitish parietal callus, while *A. atricallosus* usually has a more ovate shell, with thickened and black parietal callus. In addition, *A. leucoxanthus* tends to be distributed in the eastern part of Thailand and in Cambodia, while *A. atricallosus* occurs in eastern and southern Thailand along the Tenasserim ranges and in southern Myanmar. Moreover, molecular phylogenetic data suggested a distinct lineage between *A. leucoxanthus* and *A. atricallosus* (Sutcharit et al. 2007).

##### Amphidromus semitessellatus

Taxon classificationAnimaliaStylommatophoraCamaenidae

(Morlet, 1885)

41CDD142-CBE9-5E02-A065-AB263656D157

Fig. 11E

Bulimus (Amphidromus) semitessellatus Morlet, 1885[1884]: 387, 388, pl. 11, fig. 2, 2a. Type locality: les montagnes qui bordent le grand fleuve au delà de Stung-Treng. Les forêts et les montagnes de Kampot à Compong-Som [Mountains and forest in Stung Treng, Kampot and Sihanoukville Provinces, Cambodia].
Amphidromus (Syndromus) semitessellatus : Inkhavilay et al. 2017: 27, 28, fig. 10l, m.
Amphidromus semitessellatus : Inkhavilay et al. 2019: 94.

###### Material examined.

Locality no. 9: CUMZ-CM040 (2 shells). Locality no. 10: CUMZ-CM055 (1 shell). Locality no. 12: CUMZ-CM101 (2 shells; Fig. 11E). Locality no. 13: CUMZ-CM124 (2 shells). The empty shells were collected from the ground, and the living snails probably live on tree trunks and leaves.

###### Distribution.

Cambodia, Laos, Thailand and probably in Vietnam (Schileyko 2011, Inkhavilay et al. 2017, 2019).

###### Remarks.

This species was described based on specimens collected from the area of Kampong Som [Sihanoukville] and Kampot (Morlet 1885). Inkhavilay et al. (2017: fig. 10l) illustrated the lectotype of this species, which has a larger shell (height 35 mm) and the last whorl has only a blackish subsutural band (without any other bands on the last whorl) compared with the single worn shell that we collected from limestone near Kampot (height 23 mm), with brownish supra-peripheral and sub-peripheral bands. Thus, we provisionally identified these specimens as *A. semitessellatus* due to the similarity of both brown supra-peripheral and sub-peripheral bands on the penultimate whorls and the geographical proximity. The subgenus Syndromus typically has a small shell which exhibits high variation on shell size, colour, and pattern (see Inkhavilay et al. 2017).

#### *Trichochloritis* Pilsbry, 1891

##### Trichochloritis norodomiana

Taxon classificationAnimaliaStylommatophoraCamaenidae

(Morlet, 1883)

F49A8378-19A8-5EED-A5E4-A1D43D30DF08

Figs 5B , 11F

Helix norodomiana Morlet, 1883: 106, 107, pl. 4, fig. 3, 3a, b. Type locality: Khamchay [Cambodia].
Chloritis norodomiana : Inkhavilay et al. 2019: 102, 103, fig. 52c.

###### Material examined.

Locality no. 7: CUMZ-CM024 (2 shells). Locality no. 9: CUMZ-CM041 (35 shells). Locality no. 10: CUMZ-CM056 (1 shell). Locality no. 11: CUMZ-CM077 (4 shells; Fig. 11F), CUMZ-CM078 (1 shell). Locality no. 12: CUMZ-CM103 (1 shell). Locality no. 13: CUMZ-CM125 (4 shells), CUMZ-CM126 (1 specimen in ethanol). Locality no. 16: CUMZ-CM173 (3 specimens in ethanol; Fig. 5B). The snails were found to live on tree trunks and leaves.

###### Distribution.

Cambodia, Thailand, and Vietnam (Schileyko 2011). Schileyko (2011) reported this species from “Soutem Mt. near Xieng-Moi” as from Eastern Laos. However, “Xieng-Moi” currently refers to Chiang Mai Province in Northern Thailand.

###### Remarks.

This species was described from “Kamchay” which probably refers to Kamchay Mear, Prey Veng Province in southeastern Cambodia. The distinguishing characters of this species include a small to medium discoidal shell, periostracum thickened with short fibrous hair covering the entire shell. The spire is flat to somewhat curved with an impressed suture. The last whorl descends approaching the aperture. The peristome is circular and oblique, with narrow and thin parietal callus. The aperture opens sub-ventrally, with an expanded and whitish lip.

#### *Ganesella* Blanford, 1863

##### Ganesella perakensis

Taxon classificationAnimaliaStylommatophoraCamaenidae

(Crosse, 1879)

CC0F820D-5140-5F03-8B7B-20ADBBD3E1F4

Figs 5C , 12A

Helix (Geotrochus) perakensis Crosse, 1879: 199, 200, pl. 8, fig. 4. Type locality: Perak [Perak State, Malaysia].
Ganesella perakensis : Richardson 1985: 130. Sutcharit et al. 2019a: fig. 4d.

###### Material examined.

Locality no. 15: CUMZ-CM159 (3 shells; Fig. 12A), CUMZ-CM160 (2 shells), CUMZ-CM161 (3 specimens in ethanol; Fig. 5C). The snails were found to live on tree trunks and leaves.

###### Distribution.

Peninsula Malaysia (Sutcharit et al. 2019a).

###### Remarks.

This species was originally described from Perak, Peninsula Malaysia, and a syntype was recently figured in Sutcharit et al. (2019a: fig. 4d). Characteristics of this species are its small shell size and trochoid shape. The whorls are slightly convex, with wide and shallow suture. The shell surface exhibits thin growth lines and thin corneous periostracum. The last whorl is with well-developed peripheral keel and blunt at lower periphery. The shell colour is pale yellow to brownish, with dark brown spiral bands on peripheral keel. The apertural lip is expanded, whitish, and angled.

*Ganesella perakensis* belongs to the *G. acris* (Benson, 1859) species complex which is composed of 11 nominal species and widely distributed from Western Ghats of India to Indochina and the Greater Sunda Islands (see Richardson 1985: 129, 130). However, *G. perakensis* differs from all species known in Indochina. It differs from *G. subperakensis* (Pilsbry, 1891) from “Tonquin” and *G. vatheleti* (Bavay & Dautzenberg, 1899) from “Van Bu, Tonkin” by having a strong peripheral keel. For comparison, *G. subperakensis* is convex below periphery, with less strong peripheral keel and without brownish spiral band (Pilsbry 1891), while *G. vatheleti* exhibits a round last whorl and is more convex at base (Bavay and Dautzenberg 1899).

This species is very similar to *G. lantenoisi* (Dautzenberg & Fischer, 1906), which was described from Ha-Giang (Northern Vietnam) and Siam [Thailand]. The description itself was based mainly on the Ha-Giang specimen (Dautzenberg and Fischer 1906: pl. 9, fig. 10) while the Siamese specimen (Dautzenberg and Fischer 1906: pl. 9, fig. 11) is more similar to *G. perakensis*. Thus, the type series of this species seems to comprise of two separate species, one from northern Vietnam and one from Thailand. *Ganesella perakensis* differs from *G. lantenoisi* (specimen from Ha-Giang) in having a smaller shell (shell height up to 13 mm), shallow suture with 6 to 7 convex whorls, while *G. lantenoisi* performs an elongate trochoid, larger shell (shell height up to 18 mm), suture flattened and smooth 9 to 10 whorls. However, further investigations with both genitalia and DNA analysis will be necessary to elucidate the exact relationship between them.

##### Anceyoconcha

Taxon classificationAnimaliaStylommatophoraCamaenidae

S. Tumpeesuwan & C. Tumpeesuwan, 2020

9E87B72C-4CCB-5F6D-86FE-0B4A4924EFC2

Ganesella (Giardia) Ancey, 1907: 195, 203 (Mollusca: Eupulmonata: Camaenidae). Preoccupied by Künstler, 1882: (Metamonada: Diplomonadida: Hexamitidae).
Pseudobuliminus (Giardia) : Zilch 1960: 639.
Pseudobuliminus (Girardius) Richardson, 1983: 94. [incorrect subsequent spelling]
Giardia : Schileyko 2003: 1519, fig. 1930. Wood and Gallichan 2008: 48.
Anceyoconcha Tumpeesuwan & Tumpeesuwan in Nahok et al., 2020: 81. New replacement name.

###### Remarks.

The distinguished shell character of this genus is sinistral, elongate cylindrical to more or less conical, with 6–10 convex whorls. The last whorl is rounded (not keeled), with the aperture ovate to slightly trapezoid and the apertural lip expanded. The columella is vertical, with the umbilicus narrowly opened.

Ancey (1907) established *Giardia* as the subgenus of *Ganesella* Blanford, 1863 to include two Indochinese sinistral species: *Bulimus siamensis* Redfield, 1853 and *Bulimus rhombostomus* Pfeiffer, 1861. Subsequently, this nominal name was used as a subgeneric level of *Buliminopsis* Heude, 1890 (family Fruticicolidae) by Thiele (1931: 693). Zilch (1960: 639) transferred this nominal name to the Bradybaenidae as the subgenus of *Pseudobuliminus* Gredler, 1886, and also designated *Bulimus siamensis* Redfield, 1853 as the type species. Zilch’s classification was subsequently accepted and used by later authors (Richardson 1983, Vaught 1989). Recently, *Giardia* was treated as a valid genus under the Camaenidae (Schileyko 2003, 2011, Inkhavilay et al. 2019). However, the name *Giardia* Ancey, 1907 is a junior homonym being preoccupied by *Giardia* Künstler, 1882, a genus of anaerobic flagellated protozoan (Phylum Metamonada).

While cataloguing the land snail family Bradybaenidae, Richardson (1983) erroneously introduced the name “*Girardius*”, accompanied by diagnostic characters and attributed *Bulimus siamensis* Redfield, 1853 as the type species. However, this name is considered incorrect subsequent spelling (Schileyko 2003) and thus is not available (ICZN 1999: Art. 33.3). Nahok et al. (2020) thus proposed *Anceyoconcha* S. Tumpeesuwan & C. Tumpeesuwan, 2020 as a new name to replace *Giardia* Ancey, 1907, and included two species, *A. siamensis* and *A. rhombostoma*.

*Anceyoconcha* comprises around 15 nominal species and/or subspecies but there is an urgent need to clarify the boundary of this genus. Species and subspecies included in the genus as defined herein are: *A. chaudoensis* (Rochebrune, 1881) comb. nov., *A. maestratii* (Thach, 2017) comb. nov., *A. mantongensis* (Kobelt, 1899) comb. nov., *A. obesa* (Thach & Huber, 2018) comb. nov., *A. ovoideus* (Thach & Huber, 2018) comb. nov., *A. pharangensis* (Dautzenberg & H. Fischer, 1905) comb. nov., *A. rhombostoma pupoidea* (Dautzenberg & Fischer, 1905) comb. nov., *A. rhombostoma rhombostoma*, *A. siamensis maxima* (Ancey, 1888) comb. nov., *A. siamensis nobilis* (Ancey, 1888) comb. nov., *A. siamensis obesula* (Ancey, 1888) comb. nov., *A. siamensis pervariabilis* (Dohrn, 1863) comb. nov., *A. siamensis siamensis*, *A. siamensis zonifera* (Ancey, 1888) comb. nov. and *A. vignei* (Rochebrune, 1882) comb. nov.

The distribution of *Anceyoconcha* is probably within the Indochinese region of Cambodia, Laos, Thailand, and Vietnam (Schileyko 2011, Thach 2017, 2018, Inkhavilay et al. 2019, Nahok et al. 2020).

##### Anceyoconcha rhombostoma

Taxon classificationAnimaliaStylommatophoraCamaenidae

(Pfeiffer, 1861)

773E6D49-5BCA-5F83-8110-6C8F386BBA31

Figs 5D, E , 12C, D

Bulimus rhombostomus Pfeiffer, 1861: 194, 195. Type locality: Camboja [Cambodia].
Ganesella rhombostoma : Sutcharit et al. 2019a: 61–63, figs 1c, 3c–i, 5e–g, 7d–f.
Anceyoconcha rhombostoma : Nahok et al. 2020: 82–84, figs 2b, 3c, d, 4b, 6, 7b.

###### Material examined.

Locality no. 9: CUMZ-CM047 (66 shells), CUMZ-CM048 (5 shells; Fig. 12C), CUMZ-CM049 (9 specimens in ethanol; Fig. 5D). Locality no. 10: CUMZ-CM060 (4 shells). Locality no. 11: CUMZ-CM085 (8 shells; Fig. 12D). Locality no. 12: CUMZ-CM113 (5 shells). Locality no. 13: CUMZ-CM132 (3 shells). Locality no. 17: CUMZ-CM139 (5 shells), CUMZ-CM140 (2 specimens in ethanol; Fig. 5E). The snails were found to live on tree trunks and leaves.

###### Distribution.

Cambodia, Thailand and Vietnam (Sutcharit et al. 2019a).

###### Remarks.

This species was originally described from Cambodia based on the Mouhot collection and was recently revised by Sutcharit et al. (2019a) and Nahok et al. (2020) based on Thai and Cambodian specimens. These recent specimens were collected from Kampot area, southern Cambodia and tend to be variable in size and shape compared to the type specimens (see Sutcharit et al. (2019a: fig. 3c, d) for the lectotype and paralectotype, respectively). They have smaller shell size and an ovate trochoid shell, with a large last whorl which is well-rounded and keeled on the periphery for nearly the entire last whorl (Fig. 12C, D indicated by an arrowhead). The aperture shape is trapezoid.

There is one subspecies “Buliminus rhombostomus var. pupoidea Dautzenberg & Fischer, 1905” described from “Cochinchine: Hong-Chon” [probably in the area of south Vietnam and Cambodia]. Unfortunately, the original description was brief, without measurements and illustrations, and the type specimen could not be located. Therefore, we could not assign the southern Cambodian specimens with certainty to this subspecific entity.

##### Anceyoconcha siamensis obesula

Taxon classificationAnimaliaStylommatophoraCamaenidae

(Ancey, 1888)

ADF2BB29-F75B-52E1-8FCE-0F006E8D1E31

Fig. 12B

Buliminus siamensis var. obesula Ancey, 1888: 352. Type locality: Saigon, dans le jardin du gouverneur. Wood and Gallichan 2008: 71, pl. 6, figs 6, vi.

###### Material examined.

Locality no. 7: CUMZ-CM027 (14 shells), CUMZ-CM028 (3 shells; Fig. 12B). The empty shells were collected from the ground among leaf litter.

###### Distribution.

Known only from the type locality (Wood and Gallichan 2008).

###### Remarks.

This nominal subspecies was described from “Saigon” (see Wood and Gallichan (2008: pl. 6, fig. 6) for syntype). This species has a sinistral, ovate conic shell, having 6 to 7 whorls. The whorl is convex, having a wide and impressed suture. The shell surface is nearly smooth, with thin and brownish periostracum. The last whorl has a smaller diameter than penultimate whorl, well-rounded with weak keel near aperture. The shell colour is light brownish (becomes whitish when worn) and translucent. The aperture is subovate, with expanded and whitish lip, and thin parietal callus or thickened and whitish. The columella is straight and dilated, with a rimate umbilicus.

*Anceyoconcha siamensis obesula* differs from the nominotypical subspecies in having a much smaller shell size (shell height ranged from 15 to 20 mm), ovate shell and lower number of whorls. For comparison, *A. siamensis siamensis* (see Inkhavilay et al. 2019: fig. 53d, e) has a larger and elongate conic shell (shell height ranged from 20 to 25 mm), and with a weak keel near the aperture.

##### Anceyoconcha chaudoensis

Taxon classificationAnimaliaStylommatophoraCamaenidae

(Rochebrune, 1881)

345FACC2-57F6-5EBD-A592-13F4FB14F3B7

Figs 5F , 12E, F

Petraeus chaudoensis Rochebrune, 1881a: 70. Type locality: Montagnes de Chaudoe Cambodge [Chau Doc, An Giang Province, Vietnam].
Ena chaudocensis [sic]: Fischer 1973b: 90.

###### Material examined.

Locality no. 1: CUMZ-CM003 (20 shells; Fig. 12E, F), CUMZ-CM005 (1 specimen in ethanol; Fig. 5F). Locality no. 2: CUMZ-CM007 (9 shells). Locality no. 3: CUMZ-CM010 (1 specimen in ethanol). The snails were found to live on tree trunks and leaves.

###### Distribution.

Cambodia (Fischer 1973b).

###### Remarks.

This species was originally described from “Montagnes de Chaudoe Cambodge” [Chau Doc, An Giang Province, Vietnam] probably in the area bordering Cambodia and Vietnam. The original description of this species was brief and without illustration. This species has a sinistral elongate conic to slightly ovate conic shell, with 7 to 9 whorls, which increase regularly; cylindrical, having convex whorl and wide and impressed suture. The shell surface possesses fine growth lines, and the periostracum is thin and brownish. The last whorl is well rounded and without keel near aperture and has a similar diameter to the penultimate whorl. The shell colour is light brownish and translucent (becoming whitish when worn). The aperture is semi-ovate, with expanded and whitish lip and thin or thickened with whitish parietal callus. The columella is straight and dilated, with a rimate umbilicus.

Based on the original description, *A. chaudoensis* can be distinguished from *A. siamensis obesula* in having an elongate cylindrical shell and higher number of whorls, while the latter species has an ovate conic shell and a smaller number of whorls.

## Discussion and conclusions

Cambodia has received the least attention from malacologists for inventorying the land snail fauna, compared to other adjacent countries within the Indo-Chinese region, e.g. Thailand (Hemmen and Hemmen 2001, BEDO 2017), Laos (Inkhavilay et al. 2019) and Vietnam (Schileyko 2011). The number of species recorded in this list is relatively low, compared to that of 231 species in Laos (Inkhavilay et al. 2019), 974 species in Thailand (BEDO 2017) and 477 species (only ‘pulmonates’) in Vietnam (Schileyko 2011). It is clear that this current list represents only a small fraction of the total land snail diversity in Cambodia. Our survey did not retrieve other land snail groups which are diverse and abundant in both Thailand and Vietnam, e.g. families Alycaeidae, Clausiliidae, and Plectopylidae. It is possible that the geography of the area without high mountains and other structured habitat types result in comparatively fewer species. In comparison, most of the species Vermeulen et al. (2019a, b) reported from Southern Cambodia are small (width less than 5 mm), with the largest species being *Sesara polita* that does not exceed 12 mm in width. However, most land snails collected in our study are large (more than 10 mm) and cover most taxonomic groups, with the exception of the families Assimineidae and Diplommatinidae, both of which have been reported by Vermeulen et al. (2019a, b). The difference in taxonomic composition between Vermeulen’s and our collection probably reflect different sampling methods. More thorough investigations combining several sampling methods may uncover more land snail diversity in this area.

## Supplementary Material

XML Treatment for Georissa monterosatiana

XML Treatment for Georissa carinata

XML Treatment for Cyclophorus amoenus

XML Treatment for Cyclophorus paviei

XML Treatment for Opisthoporus bernardii

XML Treatment for Lagocheilus klobukowskii

XML Treatment for Lagocheilus landesi

XML Treatment for Pupina crosseana

XML Treatment for Valiguna siamensis

XML Treatment for Valiguna

XML Treatment for Succinea tenuis

XML Treatment for Hypselostoma benetuitum

XML Treatment for Hypselostoma cambodjense

XML Treatment for Allopeas gracile

XML Treatment for Lissachatina fulica

XML Treatment for Haploptychius

XML Treatment for Dyakia

XML Treatment for Quantula weinkauffiana

XML Treatment for Trochomorpha paviei

XML Treatment for Trochomorpha

XML Treatment for Cryptozona siamensis

XML Treatment for Hemiplecta distincta

XML Treatment for Sarika

XML Treatment for Sarika

XML Treatment for Sarika

XML Treatment for Cambodiparmarion doroshenkoi

XML Treatment for Parmarion martensi

XML Treatment for Durgella russeola

XML Treatment for Sesara

XML Treatment for Amphidromus leucoxanthus

XML Treatment for Amphidromus semitessellatus

XML Treatment for Trichochloritis norodomiana

XML Treatment for Ganesella perakensis

XML Treatment for Anceyoconcha

XML Treatment for Anceyoconcha rhombostoma

XML Treatment for Anceyoconcha siamensis obesula

XML Treatment for Anceyoconcha chaudoensis
